# Mykotoxine – Bestimmung von Aflatoxinen, Ochratoxin A, freiem Ochratoxin *α*, Gliotoxin, Citrinin und Dihydrocitrinon in Urin mittels LC-MS/MS: Biomonitoring-Methode

**DOI:** 10.34865/bi116265d10_2or

**Published:** 2025-06-30

**Authors:** Marion Berger, Max Deharde, Judith Neuhoff, Bernhard Monien, Solveigh Siodlaczek, Thomas Göen, Andrea Hartwig, MAK Commission

**Affiliations:** 1 Bundesanstalt für Arbeitsschutz und Arbeitsmedizin (BAuA). Fachbereich 4 – Gefahrstoffe und biologische Arbeitsstoffe. Gruppe 4.2 – Medizinischer Arbeitsschutz. Biomonitoring Nöldnerstraße 40/42 10317 Berlin Deutschland; 2 Bundesinstitut für Risikobewertung. Fachgruppe 54 – Abt. Lebensmittelsicherheit Max-Dohrn-Straße 8–10 10589 Berlin Deutschland; 3 Friedrich-Alexander-Universität Erlangen-Nürnberg. Institut und Poliklinik für Arbeits-, Sozial- und Umweltmedizin Henkestraße 9–11 91054 Erlangen Deutschland; 4 Institut für Angewandte Biowissenschaften. Abteilung Lebensmittelchemie und Toxikologie. Karlsruher Institut für Technologie (KIT) Adenauerring 20a, Geb. 50.41 76131 Karlsruhe Deutschland; 5 Ständige Senatskommission zur Prüfung gesundheitsschädlicher Arbeitsstoffe. Deutsche Forschungsgemeinschaft, Kennedyallee 40, 53175 Bonn, Deutschland. Weitere Informationen: Ständige Senatskommission zur Prüfung gesundheitsschädlicher Arbeitsstoffe | DFG

**Keywords:** Mykotoxine, Aflatoxine, Ochratoxin A, Gliotoxin, Citrinin, Schimmelpilzgift, Biomonitoring, Urin, LC-MS/MS, mycotoxins, aflatoxins, ochratoxin A, gliotoxin, citrinin, mould toxin, biomonitoring, urine, LC-MS/MS

## Abstract

The working group “Analyses in Biological Materials” of the German Senate Commission for the Investigation of Health Hazards of Chemical Compounds in the Work Area (MAK Commission) developed and verified the presented biomonitoring method. The aim of this method is the selective and sensitive quantitation of aflatoxins (aflatoxins B1, B2, G1, G2, M1), ochratoxin A (OTA), free ochratoxin *α *(OT*α*), gliotoxin (GT), citrinin (CIT) and dihydrocitrinone (DH‑CIT) in urine. Sample preparation comprises enrichment and purification of the analytes by solid-phase extraction using OASIS HLB cartridges. Calibration is performed with comparative standards prepared in pooled urine and treated analogously to the samples to be analysed. The aflatoxins, OTA, CIT, and DH‑CIT are quantified using isotope-labelled internal standards (ISTDs), whereas OT*α* and GT are quantified without an ISTD. Determination is carried out by high-performance liquid chromatography-tandem mass spectrometry (LC‑MS/MS). The method provides reliable and accurate analytical results, as shown by the good precision data with standard deviations below 9% for the aflatoxins, OT*α*, GT and CIT, below 13% for OTA, and below 20% for DH‑CIT. Good accuracy data were obtained with mean relative recoveries in the range of 93–107% for the aflatoxins, OT*α*, GT and CIT, in the range of 83–103% for OTA, and in the range of 81–108% for DH‑CIT. The method is both selective and sensitive, and has quantitation limits in the range of 0.013–0.022 μg/l for the aflatoxins and OTA and a quantitation limit of 1.0 μg/l for OT*α*, 1.5 μg/l for GT, 0.0075 μg/l for CIT, and 0.01 μg/l for DH‑CIT.

## 1 Kenndaten der Methode

**Table:** 

**Matrix**	Urin
**Analytisches Messprinzip**	Flüssigkeitschromatographie mit Tandem-Massenspektrometrie (LC‑MS/MS)		
**Parameter und entsprechende Arbeitsstoffe**			
**Arbeitsstoff**	**CAS‑Nr.**	**Parameter**	**CAS‑Nr.**
Aflatoxin B1 (AFB1)	1162-65-8	Aflatoxin B1 (AFB1)	1162-65-8
		Aflatoxin M1 (AFM1)	6795-23-9
Aflatoxin B2 (AFB2)	7220-81-7	Aflatoxin B2 (AFB2)	7220-81-7
Aflatoxin G1 (AFG1)	1165-39-5	Aflatoxin G1 (AFG1)	1165-39-5
Aflatoxin G2 (AFG2)	7241-98-7	Aflatoxin G2 (AFG2)	7241-98-7
Ochratoxin A (OTA)	303-47-9	Ochratoxin A (OTA)	303-47-9
		Ochratoxin α (OTα)	19165-63-0
Gliotoxin (GT)	67-99-2	Gliotoxin (GT)	67-99-2
Citrinin^a)^ (CIT)	518-75-2	Citrinin^a)^ (CIT)	518-75-2
		Dihydrocitrinona) (DH‑CIT)	65718-85-6

a) Informationen zur Bestimmung von Citrinin und Dihydrocitrinon finden sich im Anhang.

### Zuverlässigkeitskriterien

#### Aflatoxin B1 (AFB1)

**Table:** 

Präzision in der Serie:	Standardabweichung (rel.)	*s_w_* = 2,9 % bzw. 1,8 %
	Streubereich	*u* = 7,4 % bzw. 4,6 %
	bei einer dotierten Konzentration von 0,0375 μg oder 0,13 μg AFB1 pro Liter Urin und n = 6 Bestimmungen	
Präzision von Tag zu Tag:	Standardabweichung (rel.)	*s_w_* = 5,2 % bzw. 3,6 %
	Streubereich	*u* = 13,4 % bzw. 8,6 %
	bei einer dotierten Konzentration von 0,0375 μg oder 0,13 μg AFB1 pro Liter Urin und n = 6 oder 8 Bestimmungen	
Richtigkeit in der Serie:	Wiederfindung (rel.)	*r* = 98,8 % bzw. 100 %
	bei einer dotierten Konzentration von 0,0375 μg oder 0,13 μg AFB1 pro Liter Urin und n = 6 Bestimmungen	
Richtigkeit von Tag zu Tag:	Wiederfindung (rel.)	*r* = 106 % bzw. 93,5 %
	bei einer dotierten Konzentration von 0,0375 μg oder 0,13 μg AFB1 pro Liter Urin und n = 6 oder 8 Bestimmungen	
Nachweisgrenze:	0,004 μg AFB1 pro Liter Urin
Bestimmungsgrenze:	0,013 μg AFB1 pro Liter Urin

#### Aflatoxin B2 (AFB2)

**Table:** 

Präzision in der Serie:	Standardabweichung (rel.)	*s_w_* = 2,4 % bzw. 2,2 %
	Streubereich	*u* = 6,2 % bzw. 5,6 %
	bei einer dotierten Konzentration von 0,0654 μg oder 0,22 μg AFB2 pro Liter Urin und n = 6 Bestimmungen	
Präzision von Tag zu Tag:	Standardabweichung (rel.)	*s_w_* = 2,2 % bzw. 3,6 %
	Streubereich	*u* = 5,6 % bzw. 8,5 %
	bei einer dotierten Konzentration von 0,0654 μg oder 0,22 μg AFB2 pro Liter Urin und n = 6 oder 8 Bestimmungen	
Richtigkeit in der Serie:	Wiederfindung (rel.)	*r* = 104 % bzw. 102 %
	bei einer dotierten Konzentration von 0,0654 μg oder 0,22 μg AFB2 pro Liter Urin und n = 6 Bestimmungen	
Richtigkeit von Tag zu Tag:	Wiederfindung (rel.)	*r* = 103 % bzw. 95,7 %
	bei einer dotierten Konzentration von 0,0654 μg oder 0,22 μg AFB2 pro Liter Urin und n = 6 oder 8 Bestimmungen	
Nachweisgrenze:	0,007 μg AFB2 pro Liter Urin
Bestimmungsgrenze:	0,022 μg AFB2 pro Liter Urin

#### Aflatoxin G1 (AFG1)

**Table:** 

Präzision in der Serie:	Standardabweichung (rel.)	*s_w_* = 3,5 % bzw. 2,6 %
	Streubereich	u = 9,1 % bzw. 6,8 %
	bei einer dotierten Konzentration von 0,0375 μg oder 0,13 μg AFG1 pro Liter Urin und n = 6 Bestimmungen	
Präzision von Tag zu Tag:	Standardabweichung (rel.)	*s_w_* = 4,9 % bzw. 3,5 %
	Streubereich	u = 12,5 % bzw. 8,3 %
	bei einer dotierten Konzentration von 0,0375 μg oder 0,13 μg AFG1 pro Liter Urin und n = 6 oder 8 Bestimmungen	
Richtigkeit in der Serie:	Wiederfindung (rel.)	r = 99,4 % bzw. 100 %
	bei einer dotierten Konzentration von 0,0375 μg oder 0,13 μg AFG1 pro Liter Urin und n = 6 Bestimmungen	
Richtigkeit von Tag zu Tag:	Wiederfindung (rel.)	r = 107 % bzw. 94,6 %
	bei einer dotierten Konzentration von 0,0375 μg oder 0,13 μg AFG1 pro Liter Urin und n = 6 oder 8 Bestimmungen	
Nachweisgrenze:	0,004 μg AFG1 pro Liter Urin
Bestimmungsgrenze:	0,013 μg AFG1 pro Liter Urin

#### Aflatoxin G2 (AFG2)

**Table:** 

Präzision in der Serie:	Standardabweichung (rel.)	*s_w_* = 2,0 % bzw. 3,2 %
	Streubereich	*u* = 5,0 % bzw. 8,3 %
	bei einer dotierten Konzentration von 0,163 μg oder 0,54 μg AFG2 pro Liter Urin und n = 6 Bestimmungen	
Präzision von Tag zu Tag:	Standardabweichung (rel.)	*s_w_* = 2,7 % bzw. 3,3 %
	Streubereich	*u* = 7,0 % bzw. 7,8 %
	bei einer dotierten Konzentration von 0,163 μg oder 0,54 μg AFG2 pro Liter Urin und n = 6 oder 8 Bestimmungen	
Richtigkeit in der Serie:	Wiederfindung (rel.)	*r* = 100 % bzw. 103 %
	bei einer dotierten Konzentration von 0,163 μg oder 0,54 μg AFG2 pro Liter Urin und n = 6 Bestimmungen	
Richtigkeit von Tag zu Tag:	Wiederfindung (rel.)	*r* = 95,8 % bzw. 96,3 %
	bei einer dotierten Konzentration von 0,163 μg oder 0,54 μg AFG2 pro Liter Urin und n = 6 oder 8 Bestimmungen	
Nachweisgrenze:	0,02 μg AFG2 pro Liter Urin
Bestimmungsgrenze:	0,054 μg AFG2 pro Liter Urin

#### Aflatoxin M1 (AFM1)

**Table:** 

Präzision in der Serie:	Standardabweichung (rel.)	*s_w_* = 3,1 % bzw. 3,6 %
	Streubereich	*u* = 7,9 % bzw. 9,4 %
	bei einer dotierten Konzentration von 0,066 μg oder 0,22 μg AFM1 pro Liter Urin und n = 6 Bestimmungen	
Präzision von Tag zu Tag:	Standardabweichung (rel.)	*s_w_* = 3,6 % bzw. 5,9 %
	Streubereich	*u* = 9,3 % bzw. 13,9 %
	bei einer dotierten Konzentration von 0,066 μg oder 0,22 μg AFM1 pro Liter Urin und n = 6 oder 8 Bestimmungen	
Richtigkeit in der Serie:	Wiederfindung (rel.)	*r* = 108 % bzw. 101 %
	bei einer dotierten Konzentration von 0,066 μg oder 0,22 μg AFM1 pro Liter Urin und n = 6 Bestimmungen	
Richtigkeit von Tag zu Tag:	Wiederfindung (rel.)	*r* = 103 % bzw. 96,4 %
	bei einer dotierten Konzentration von 0,066 μg oder 0,22 μg AFM1 pro Liter Urin und n = 6 oder 8 Bestimmungen	
Nachweisgrenze:	0,007 μg AFM1 pro Liter Urin
Bestimmungsgrenze:	0,022 μg AFM1 pro Liter Urin

#### Ochratoxin A (OTA)

**Table:** 

Präzision in der Serie:	Standardabweichung (rel.)	*s_w_* = 1,5 % bzw. 3,6 %
	Streubereich	*u* = 3,9 % bzw. 9,4 %
	bei einer dotierten Konzentration von 0,0377 μg oder 0,13 μg OTA pro Liter Urin und n = 6 Bestimmungen	
Präzision von Tag zu Tag:	Standardabweichung (rel.)	*s_w_* = 8,8 % bzw. 12,5 %
	Streubereich	*u* = 22,7 % bzw. 30,6 %
	bei einer dotierten Konzentration von 0,0377 μg oder 0,13 μg OTA pro Liter Urin und n = 6 oder 7 Bestimmungen	
Richtigkeit in der Serie:	Wiederfindung (rel.)	*r* = 103 % bzw. 93,5 %
	bei einer dotierten Konzentration von 0,0377 μg oder 0,13 μg OTA pro Liter Urin und n = 6 Bestimmungen	
Richtigkeit von Tag zu Tag:	Wiederfindung (rel.)	*r* = 92,5 % bzw. 82,9 %
	bei einer dotierten Konzentration von 0,0377 μg oder 0,13 μg OTA pro Liter Urin und n = 6 oder 7 Bestimmungen	
Nachweisgrenze:	0,004 μg OTA pro Liter Urin
Bestimmungsgrenze:	0,013 μg OTA pro Liter Urin

#### Ochratoxin α (OTα)

**Table:** 

Präzision in der Serie:	Standardabweichung (rel.)	*s_w_* = 1,3 % bzw. 4,0 %
	Streubereich	u = 3,3 % bzw. 10,4 %
	bei einer dotierten Konzentration von 2,5 μg oder 6,25 μg OTα pro Liter Urin und n = 6 Bestimmungen	
Präzision von Tag zu Tag:	Standardabweichung (rel.)	*s_w_* = 5,2 % bzw. 6,5 %
	Streubereich	u = 13,2 % bzw. 15,3 %
	bei einer dotierten Konzentration von 2,5 μg oder 6,25 μg OTα pro Liter Urin und n = 6 oder 8 Bestimmungen	
Richtigkeit in der Serie:	Wiederfindung (rel.)	r = 95,4 % bzw. 93,4 %
	bei einer dotierten Konzentration von 2,5 μg oder 6,25 μg OTα pro Liter Urin und n = 6 Bestimmungen	
Richtigkeit von Tag zu Tag:	Wiederfindung (rel.)	r = 99,9 % bzw. 99,5 %
	bei einer dotierten Konzentration von 2,5 μg oder 6,25 μg OTα pro Liter Urin und n = 6 oder 8 Bestimmungen	
Nachweisgrenze:	0,4 μg OTα pro Liter Urin
Bestimmungsgrenze:	1,0 μg OTα pro Liter Urin

#### Gliotoxin (GT)

**Table:** 

Präzision in der Serie:	Standardabweichung (rel.)	*s_w_* = 1,7 % bzw. 4,9 %
	Streubereich	*u* = 4,4 % bzw. 12,7 %
	bei einer dotierten Konzentration von 3,75 μg oder 8,75 μg GT pro Liter Urin und n = 6 Bestimmungen	
Präzision von Tag zu Tag:	Standardabweichung (rel.)	*s_w_* = 6,6 % bzw. 8,6 %
	Streubereich	*u* = 16,9 % bzw. 20,4 %
	bei einer dotierten Konzentration von 3,75 μg oder 8,75 μg GT pro Liter Urin und n = 6 oder 8 Bestimmungen	
Richtigkeit in der Serie:	Wiederfindung (rel.)	*r* = 95,4 % bzw. 92,9 %
	bei einer dotierten Konzentration von 3,75 μg oder 8,75 μg GT pro Liter Urin und n = 6 Bestimmungen	
Richtigkeit von Tag zu Tag:	Wiederfindung (rel.)	*r* = 97,1 % bzw. 104 %
	bei einer dotierten Konzentration von 3,75 μg oder 8,75 μg GT pro Liter Urin und n = 6 oder 8 Bestimmungen	
Nachweisgrenze:	0,5 μg GT pro Liter Urin
Bestimmungsgrenze:	1,5 μg GT pro Liter Urin

#### Citrinin (CIT)^a)^

**Table:** 

Präzision in der Serie:	Standardabweichung (rel.)	*s_w_* = 3,1 %, 6,0 % bzw. 6,5 %
	Streubereich	*u* = 9,4 %, 16,5 % bzw. 17,2 %
	bei einer dotierten Konzentration von 0,01 μg, 0,1 μg oder 1,0 μg CIT pro Liter Urin und n = 6 Bestimmungen	
Präzision von Tag zu Tag:	Standardabweichung (rel.)	*s_w_* = 6,4 %, 5,9 % bzw. 2,1 %
	Streubereich	*u* = 17,5 % 15,2 % bzw. 6,2 %
	bei einer dotierten Konzentration von 0,01 μg, 0,1 μg oder 1,0 μg CIT pro Liter Urin und n = 6 Bestimmungen	
Richtigkeit in der Serie:	Wiederfindung (rel.)	*r* = 94,1 %, 88,6 % bzw. 93,3 %
	bei einer dotierten Konzentration von 0,01 μg, 0,1 μg oder 1,0 μg CIT pro Liter Urin und n = 6 Bestimmungen	
Richtigkeit von Tag zu Tag:	Wiederfindung (rel.)	*r* = 99,8 %, 99,0 % bzw. 98,5 %
	bei einer dotierten Konzentration von 0,01 μg, 0,1 μg oder 1,0 μg CIT pro Liter Urin und n = 6 Bestimmungen	
Nachweisgrenze:	0,0003 μg CIT pro Liter Urin
Bestimmungsgrenze:	0,001 μg CIT pro Liter Urin	

a) Diese Daten wurden von den Prüfern der Methode erhoben. Angaben zur Bestimmung von Citrinin und Dihydrocitrinon finden sich im Anhang.

#### Dihydrocitrinon (DH‑CIT)^a)^

**Table:** 

Präzision in der Serie:	Standardabweichung (rel.)	*s_w_* = 14,0 %, 3,3 % bzw. 8,9 %
	Streubereich	*u* = 37,5 %, 10,1 % bzw. 23,9 %
	bei einer dotierten Konzentration von 0,01 μg, 0,1 μg oder 1,0 μg DH-CIT pro Liter Urin und n = 6 Bestimmungen	
Präzision von Tag zu Tag:	Standardabweichung (rel.)	*s_w_* = 19,3 %, 5,4 % bzw. 3,4 %
	Streubereich	*u* = 54,7 %, 12,1 % bzw. 9,9 %
	bei einer dotierten Konzentration von 0,01 μg, 0,1 μg oder 1,0 μg DH-CIT pro Liter Urin und n = 6 Bestimmungen	
Richtigkeit in der Serie:	Wiederfindung (rel.)	*r* = 108 %, 80,9 % bzw. 88,5 %
	bei einer dotierten Konzentration von 0,01 μg, 0,1 μg oder 1,0 μg DH-CIT pro Liter Urin und n = 6 Bestimmungen	
Richtigkeit von Tag zu Tag:	Wiederfindung (rel.)	*r* = 108 %, 88,4 % bzw. 96,4 %
	bei einer dotierten Konzentration von 0,01 μg, 0,1 μg oder 1,0 μg DH-CIT pro Liter Urin und n = 6 Bestimmungen	
Nachweisgrenze:	0,0075 μg DH-CIT pro Liter Urin
Bestimmungsgrenze:	0,01 μg DH-CIT pro Liter Urin	

a) Diese Daten wurden von den Prüfern der Methode erhoben. Angaben zur Bestimmung von Citrinin und Dihydrocitrinon finden sich im Anhang.

## 2 Allgemeine Informationen zu den Mykotoxinen

**Abb.1:**
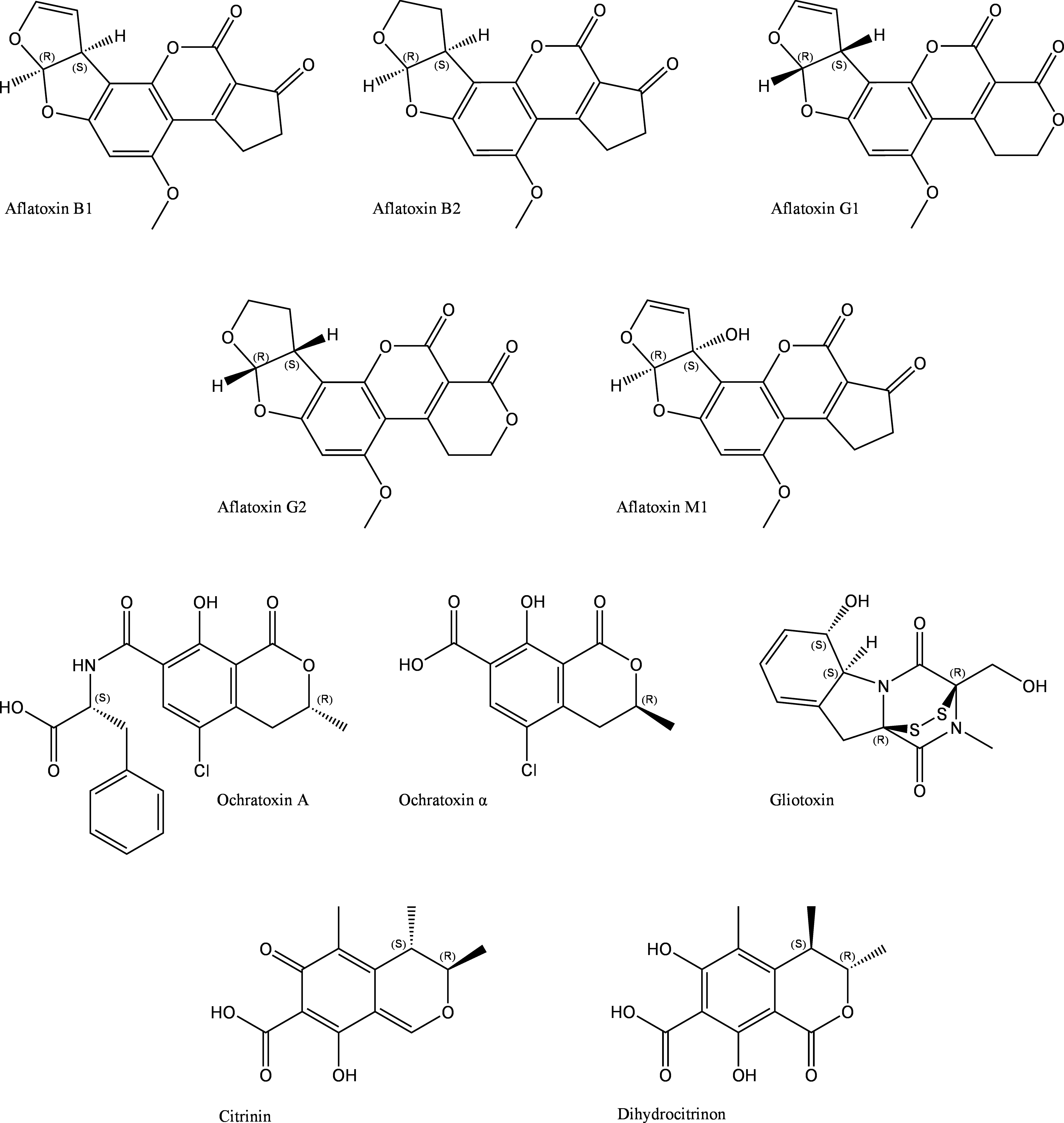
Strukturformeln von Aflatoxin B1, Aflatoxin B2, Aflatoxin G1, Aflatoxin G2, Aflatoxin M1, Ochratoxin A, Ochratoxin α, Gliotoxin, Citrinin sowie Dihydrocitrinon

Die Strukturformeln der mit dieser Methode quantifizierbaren Mykotoxine und ihrer Metaboliten sind in Abbildung 1 dargestellt. Eine Methode zur Bestimmung weiterer Mykotoxine im Urin (Deoxynivalenol und Deepoxydeoxy­nivalenol) wurde von der Kommission publiziert (Berger et al. 2025).

### Aflatoxine

Die natürlich vorkommenden Aflatoxine AFB1, AFB2, AFG1 und AFG2 sind Bisfuranocumarinverbindungen, die von verschiedenen Schimmelpilzen der Gattung *Aspergillus*, insbesondere *A. flavus* und *A. parasiticus*, produziert werden (CONTAM et al. 2020).

Aflatoxine wirken beim Menschen genotoxisch und krebserzeugend (CONTAM et al. 2020). Die Kommission hat Aflatoxine in die Kanzerogenitäts-Kategorie 1 und in die Keimzellmutagenitäts-Kategorie 3A eingestuft (Greim 2008).

Aflatoxine können sowohl oral als auch inhalativ aufgenommen werden (Greim 2008). In‑vitro-Experimente zeigen für AFB1 zudem eine geringe dermale Penetrationsfähigkeit (Boonen et al. 2012). Toxikokinetische Daten aus Humanexperimenten liegen ausschließlich für AFB1 vor. Nach oraler Aufnahme wird AFB1 rasch über den Gastrointestinaltrakt resorbiert, wobei maximale Konzentrationen im Blutplasma innerhalb einer Stunde erreicht werden (Jubert et al. 2009). Für AFB1 wird eine zweiphasige Kinetik beobachtet: einer schnellen Verteilungs- und Ausscheidungsphase mit einer Plasmahalbwertszeit von ca. 2,9 h folgt eine langsame Ausscheidungsphase mit einer Plasmahalbwertszeit von ca. 64 h. 95 % des über den Urin ausgeschiedenen AFB1 wird innerhalb eines Tages ausgeschieden (Jubert et al. 2009). Aflatoxine werden hauptsächlich in der Leber metabolisiert (Al-Jaal et al. 2019), wobei für AFB1 vier Hauptstoffwechselpfade bekannt sind: Demethylierung zu Aflatoxin P1, Ketoreduktion zu Aflatoxicol, Hydroxylierung zu AFM1 und Epoxidierung zum AFB1-8,9‑Epoxid (Dohnal et al. 2014). Die durch Oxidierung der Furandoppelbindung gebildeten 8,9‑Epoxide von AFB1 und AFG1 können mit der DNA und anderen Nukleophilen rea­gieren (CONTAM et al. 2020). AFB1 bildet dabei kovalente DNA‑Addukte mit N7‑Guanin und verursacht DNA‑Läsionen (CONTAM et al. 2020).

Die Allgemeinbevölkerung nimmt Aflatoxine durch den Verzehr belasteter Lebensmittel (u. a. von Erdnüssen und Gewürzen) auf (CONTAM et al. 2020). Berufliche Expositionen sind für die Landwirtschaft und Lebensmittelproduktion sowie für die Abfallentsorgung beschrieben (Brera et al. 2002; Ferri et al. 2017; Fromme et al. 2016; Viegas et al. 2013, 2015, 2018 a).

Als Urinbiomarker einer Aflatoxinexposition werden neben den Aflatoxinen selbst (AFB1, AFB2, AFG1 und AFG2) der Metabolit AFM1 und das Guanin-Addukt 8,9‑Dihydro-8‑(N7‑guanyl)-9‑hydroxy-AFB1 (AFB1-N7‑Gua) verwendet (Al-Jaal et al. 2019). Dabei gelten AFM1 und AFB1-N7‑Gua als valide Biomarker einer erst kurz zurückliegenden Aflatoxinexposition (CONTAM et al. 2020). Aufgrund der Verfügbarkeit analytischer Standards wird AFM1 häufiger als Biomarker verwendet (Martins et al. 2021).

### Ochratoxin A (OTA)

OTA wird von Schimmelpilzen der Gattungen *Aspergillus* und *Penicillium* gebildet und besitzt krebserzeugende, nephrotoxische, reproduktionstoxische und immuntoxische Eigenschaften (Tao et al. 2018). Die Kommission hat OTA in die Kanzerogenitäts-Kategorie 2 und in die Keimzellmutagenitäts-Kategorie 3 B eingestuft (Greim 2003).

Oral aufgenommenes OTA wird gut über den Gastrointestinaltrakt resorbiert und anschließend nahezu vollständig an Serumproteine, wie Albumin, gebunden. Der Anteil ungebundenen OTAs im Blut ist mit 0,02 % gering (Hagelberg et al. 1989).

Die Exkretion erfolgt beim Menschen hauptsächlich über die Nieren. Aufgrund der starken Proteinbindung findet eine glomeruläre Filtration nur in begrenztem Umfang statt und OTA gelangt stattdessen durch tubuläre Sekretion in den Harn. In allen Segmenten des Nephrons wird OTA rückresorbiert, wodurch eine Akkumulation in der Niere erfolgen kann (HBM-Kommission 2014).

Sowohl im Tierversuch als auch beim Menschen (n = 1) wird eine zweiphasige Toxikokinetik beobachtet, wobei einer schnellen Verteilungs- und Ausscheidungsphase mit einer Plasmahalbwertszeit von ca. 20 h eine langsame Aus­scheidungsphase mit einer Plasmahalbwertszeit von ca. 35 Tagen folgt (Studer-Rohr et al. 2000). Etwa 50 % des aufgenommenen OTAs werden unmetabolisiert ausgeschieden. OTA wird in Nieren, Leber und Darm verstoffwechselt. Hauptmetabolit ist OTα, das zum Teil auch als Glucuronid und/oder Sulfatester vorliegt (Muñoz et al. 2010).

Die Allgemeinbevölkerung nimmt OTA durch den Verzehr belasteter Lebensmittel (u. a. von Kaffee und Getreide­produkten) auf. Berufliche Expositionen sind für die Landwirtschaft und Lebensmittelproduktion (Brera et al. 2002; Fromme et al. 2016; Viegas et al. 2019) sowie für die Abfallentsorgung (Degen et al. 2003; Viegas et al. 2018 b) beschrieben.

Aufgrund der langen Plasmahalbwertszeit werden chronische Expositionen (über mehrere Wochen) durch eine Bestimmung der OTA‑Konzentration im Serum/Plasma erfasst. OTA‑Konzentrationen im Urin hingegen bilden akute Expositionen besser ab (Duarte et al. 2011; EFSA 2006).

### Gliotoxin (GT)

GT ist ein schwefelhaltiges Mykotoxin, das zur Gruppe der Epipolythiodioxopiperazine gehört und von verschiedenen Schimmelpilzen, u. a. *Aspergillus fumigatus*, *Eurotium chevalieri*, *Trichoderma virens* und *Neosartorya pseudo­fischeri*, gebildet wird (Scharf et al. 2016). GT wirkt immunsuppressiv, genotoxisch und zytotoxisch (Nieminen et al. 2002 a, b). Darüber hinaus steht es unter Verdacht einen Einfluss auf die Virulenz von *A. fumigatus* zu haben und dadurch die Ausbildung einer invasiven Aspergillose zu fördern (Hof und Kupfahl 2009). Gliotoxin wurde noch nicht von der Kommission bewertet.

Über Toxikokinetik und den Metabolismus im menschlichen Körper liegen kaum Informationen vor. De Santis et al. (2017) konnten in 71,6 % der Urinproben von Kindern GT mit einer maximalen Konzentration von 114,7 μg/l bestimmen. Darüber hinaus wird der Einsatz von GT als Biomarker in Serum- und Urinproben zur Früherkennung von invasiver Aspergillose diskutiert (Cerqueira et al. 2014; Gao et al. 2019).

Hohe Konzentrationen des ubiquitär auftretenden *A. fumigatus* werden u. a. in Bioaerosolproben in der Bioabfall­behandlung beobachtet (Fischer et al. 2000). Nur wenige Studien haben GT in Luft- und Staubproben im landwirtschaftlichen Sektor untersucht. In Staubproben aus Getreidelagern lagen GT-Konzentrationen von bis zu 319,6 μg/g vor (Tangni und Pussemier 2007). Lanier et al. (2010) berichten von Stallluftkonzentrationen von bis zu 3,7 μg/m^3^ bei der Verfütterung von Maissilage, Heu und Ölsaaten.

Systematische Untersuchungen über die Verunreinigung von Lebensmitteln mit GT und zur ernährungsbedingten Hintergrundbelastung liegen nicht vor (Scharf et al. 2016).

### Citrinin (CIT)

CIT ist ein Polyketid, das von Pilzen als sekundäres Stoffwechselprodukt gebildet wird und nach *Penicillium citrinum* benannt ist, aus dem es erstmals isoliert wurde. CIT wird von verschiedenen Spezies der Gattungen *Penicillium*, *Aspergillus*, sowie *Monascus* gebildet und kann in unterschiedlichen Klimazonen nachgewiesen werden (CONTAM 2012).

CIT wird häufig zusammen mit dem strukturell und toxikologisch ähnlichem OTA gefunden. Die Niere ist bei verschiedenen Säugetierspezies das primäre Zielorgan beider Mykotoxine (CONTAM 2012; EFSA 2006). Die International Agency for Research on Cancer (IARC) hat CIT – wegen eines Verdachts auf Kanzerogenität bei Ratten und unzu­reichender Beweise beim Menschen – in Gruppe 3 eingestuft (nicht klassifizierbar hinsichtlich der Human­kanzero­genität) (IARC 1986). Die Europäische Behörde für Lebensmittelsicherheit (European Food Safety Authority; EFSA) konnte das mögliche kanzerogene Potential von CIT nicht bewerten (CONTAM 2012). CIT wurde bisher noch nicht von der Kommission bewertet.

CIT kommt vor allem in gelagertem Getreide und Getreideprodukten vor, aber auch in anderen mit Schimmelpilzen belas­teten pflanzlichen Produkten, wie Früchten, Kräutern und Gewürzen. CIT entsteht zumeist während der Lagerung, wobei Konzentrationen bis zu 1500 μg/kg nachgewiesen wurden (CONTAM 2012). Sehr hohe Gehalte an CIT (> 2000 μg/kg) wurden in Rotschimmelreis nachgewiesen, der als Konservierungsmittel und Farbstoff in asiatischen Lebensmitteln verwendet und als Nahrungsergänzungsmittel vermarktet wird (Degen et al. 2022).

CIT wird vor allem mit der Nahrung aufgenommen, aber eine dermale (Boonen et al. 2012) und inhalative (Föllmann et al. 2016) Aufnahme scheint ebenfalls möglich zu sein. Untersuchungen zur CIT-Kinetik beim Menschen zeigen nach oraler Aufnahme eine schnelle Resorption und eine mittlere Halbwertszeit im Blutplasma von 9,4 h (Degen et al. 2018). CIT wird weitgehend zu DH-CIT verstoffwechselt, das zusammen mit der nicht metabolisierten Verbindung mit dem Urin ausgeschieden wird. Die mittleren Exkretions-Halbwertszeiten von CIT und DH-CIT im Urin betragen 6,7 h bzw. 8,9 h. Dabei werden etwa 40 % (Summe von CIT und DH-CIT) der aufgenommenen Menge innerhalb von 24 h ausgeschieden (Degen et al. 2018).

## 3 Grundlage des Verfahrens

Das hier beschriebene Verfahren dient der Erfassung von Aflatoxinen (AFB1, AFB2, AFG1, AFG2, AFM1), OTA, freiem OTα und GT in Humanurin mittels LC‑MS/MS. Die Probenvorbereitung umfasst die Aufreinigung der Proben mittels Festphasenextraktion an OASIS‑HLB-Kartuschen gefolgt von einer Aufkonzentrierung der Eluate im Stickstoffstrom. Die Kalibrierung erfolgt mit Vergleichsstandards, die in Urin angesetzt und in der gleichen Weise behandelt werden wie die zu analysierenden Proben. Die Quantifizierung erfolgt für die Aflatoxine mit ^13^C_17_‑AFB1, für OTA mit ^13^C_20_‑OTA und für OTα und GT ohne ISTD.

Bei der Methodenprüfung wurden zusätzlich CIT und dessen Metabolit DH-CIT in die Methode integriert. Infor­mationen zur Bestimmung dieser Parameter und die entsprechenden Validierungsdaten finden sich im Anhang.

## 4 Geräte, Chemikalien und Lösungen

### 4.1 Geräte

- HPLC‑Anlage mit binärer Pumpe, Autosampler, Säulenofen und Degasser (z. B. Nexera XR, Shimadzu Deutschland GmbH, Duisburg)
- Triple-Quadrupol-Massenspektrometer (z. B. AB SCIEX QTRAP 5500, AB SCIEX Germany GmbH, Darmstadt)
- Analytische HPLC‑Trennsäule (z. B. Kinetex^®^ Core-Shell Technologie; Kinetex^®^ 2,6 μm Biphenyl 100 Å, 100 × 2,1 mm, Phenomenex Ltd. Deutschland, Aschaffenburg)
- UHPLC‑Vorsäule (z. B. Nr. AJO-9209, SecurityGuard ULTRA Cartridges, Biphenyl 2,1 mm ID, inklusive Säulenhalter, Phenomenex Ltd. Deutschland, Aschaffenburg)
- Stickstoffgenerator (z. B. cmc Instruments GmbH, Eschborn)
- Reinstwasseranlage (z. B. Veolia Water Solutions & Technologies, Saint-Maurice, Frankreich)
- Laborzentrifuge (z. B. Fisher Scientific GmbH, Schwerte)
- Analysenwaage (z. B. Sartorius AG, Göttingen)
- pH‑Meter (z. B. Mettler-Toledo GmbH, Gießen)
- Abblasstation (z. B. Biotage Sweden AB, Uppsala, Schweden)
- Ultraschallbad zum Entgasen der Eluenten (z. B. SONOREX SUPER RK 510 H, BANDELIN electronic GmbH & Co. KG, Berlin)
- Rotationsmischer (z. B. Cole-Parmer^TM^ Stuart^TM^, Fisher Scientific GmbH, Schwerte)
- Vortex-Schüttler (z. B. IKA‑Werke GmbH & Co. KG, Staufen)
- Vorrichtung für die Festphasenextraktion (z. B. VisiPrep^TM^ SPE‑Vakuumverteiler, Supelco^®^, Merck KGaA, Darmstadt)
- Variabel einstellbare Pipetten mit passenden Pipettenspitzen (z. B. Eppendorf AG, Hamburg)
- 2500‑ml‑Glasflaschen mit Schraubverschluss (z. B. DURAN^®^, Schott AG, Mainz)
- Verschiedene Messkolben und Bechergläser (z. B. DURAN^®^, Schott AG, Mainz)
- 10‑ml‑Braunglasflaschen (z. B. DURAN^®^, Schott AG, Mainz)
- 0,2‑μm‑Spritzenvorsatzfilter (13 mm, regenerierte Zellulose) (z. B. CS – Chromatographie Service GmbH, Langerwehe)
- SPE‑Kartuschen, OASIS^®^ HLB, 150 mg/6 ml (z. B. Waters GmbH, Eschborn)
- 15‑ml‑Polypropylen‑Zentrifugenröhrchen mit konischem Boden (z. B. COTECH Vertriebs GmbH, Berlin)
- 13‑ml‑Polypropylen‑Zentrifugenröhrchen mit rundem Boden (z. B. COTECH Vertriebs GmbH, Berlin)
- 5‑ml‑Luer-Lock-Einmalspritzen mit Einmal-Injektionskanülen (z. B. Omnifix^®^ Luer Solo, B. Braun SE, Melsungen)
- 1,5‑ml‑Polypropylen‑Gewindefläschchen mit Schraubkappen (z. B. MACHEREY-NAGEL GmbH & Co. KG, Düren)
- Urinbecher aus Polypropylen (z. B. Sarstedt AG & Co. KG, Nümbrecht)

### 4.2 Chemikalien

Wenn nicht anders angegeben, sind alle genannten Chemikalien mindestens in p. a.‑Qualität zu verwenden.

- Acetonitril, LC‑MS (z. B. Nr. 15037, Burdick & Jackson^TM^, Honeywell International Inc., Morristown, USA)
- Ammoniumacetat (z. B. Nr. 15681570, Honeywell Fluka^TM^, Fisher Scientific GmbH, Schwerte)
- Essigsäure, LiChropur^®^, 100 % (z. B. Nr. 533001, Supelco^®^, Merck KGaA, Darmstadt)
- Isopropanol, LiChrosolv^®^, für die Hinterkolbenspülung der Pumpen (z. B. Nr. 102781, Supelco^®^, Merck KGaA, Darmstadt)
- Methanol, LiChrosolv^®^, ≥ 99,97 % (z. B. Nr. 106035, Supelco^®^, Merck KGaA, Darmstadt)
- Salzsäure, ROTIPURAN^®^, 37 % (z. B. Nr. 4625.1, Carl Roth GmbH + Co. KG, Karlsruhe)
- Hochreines Wasser (z. B. Veolia Water Solutions & Technologies, Saint-Maurice, Frankreich)
- Nativer Urin von Freiwilligen mit möglichst geringen Mykotoxin-Hintergrundgehalten

### 4.3 Referenzstandards und ISTDs

- Aflatoxin B1, 2 μg/ml in Acetonitril (z. B. Romer Labs Division Holding GmbH, Getzersdorf, Österreich)
- ^13^C_17_‑Aflatoxin B1, 0,501 μg/ml in Acetonitril (z. B. Nr. DRE‑A10047150AL‑0.5, LGC Standards GmbH, Wesel)
- Aflatoxin B2 (z. B. Nr. A9887, Sigma-Aldrich^®^, Merck KGaA, Darmstadt)
- Aflatoxin G1, 2 μg/ml in Acetonitril (z. B. Romer Labs Division Holding GmbH, Getzersdorf, Österreich)
- Aflatoxin G2, 0,51 μg/ml in Acetonitril (z. B. Romer Labs Division Holding GmbH, Getzersdorf, Österreich)
- Aflatoxin M1, 0,506 μg/ml in Acetonitril (z. B. Romer Labs Division Holding GmbH, Getzersdorf, Österreich)
- Ochratoxin A, 10,05 μg/ml in Acetonitril (z. B. Romer Labs Division Holding GmbH, Getzersdorf, Österreich)
- ^13^C_20_‑Ochratoxin A, 10,10 μg/ml in Acetonitril (z. B. Romer Labs Division Holding GmbH, Getzersdorf, Österreich)
- Gliotoxin (z. B. Nr. G9893, Sigma-Aldrich^®^, Merck KGaA, Darmstadt)
- Ochratoxin α, 10,2 μg/ml in Acetonitril (z. B. Romer Labs Division Holding GmbH, Getzersdorf, Österreich)

### 4.4 Lösungen

- Eluent A
  In einen 1000-ml-Messkolben werden 77,08 mg Ammoniumacetat eingewogen und in ein wenig hochreinem Wasser gelöst. Anschließend wird 1 ml Essigsäure in den Kolben pipettiert und dieser mit hochreinem Wasser bis zur Markierung aufgefüllt.
- Eluent B
  In einen 1000-ml-Messkolben werden 77,08 mg Ammoniumacetat eingewogen und in ein wenig Methanol gelöst. Anschließend wird 1 ml Essigsäure in den Kolben pipettiert und dieser mit Methanol bis zur Markierung aufgefüllt.
- Gradientenlösung (Eluent A ∶ Eluent B; 98 ∶ 2 (V : V))
  2 ml Eluent B werden in einen 100‑ml-Messkolben pipettiert, anschließend wird der Messkolben mit Eluent A bis zur Markierung aufgefüllt.
- verdünnte Salzsäure (55 mM)
  In einem 1000-ml-Messkolben werden ca. 550 ml hochreines Wasser vorgelegt und 4,56 ml der 37%igen Salzsäure dazugegeben. Anschließend wird der Messkolben mit hochreinem Wasser bis zur Markierung aufgefüllt.
- Methanol (2 % in hochreinem Wasser)
  In einem 100-ml-Messkolben werden 2 ml Methanol vorgelegt. Anschließend wird der Messkolben mit hochreinem Wasser bis zur Markierung aufgefüllt.

Die Lösungen werden bei Raumtemperatur gelagert.

### 4.5 Interner Standard (ISTD)

- ISTD-Dotierlösung (ISTD-DL; 30,12 μg ^13^C_17_‑AFB1/l und 30,3 μg ^13^C_20_‑OTA/l)
  In einem 1,5-ml-Polypropylengewindefläschchen werden 60 μl des ^13^C_17_‑AFB1‑Standards und 3 μl des ^13^C_20_‑OTA‑­Standards mit 937 μl Acetonitril gemischt.

Die ISTD-Dotierlösung wird bei −20 °C gelagert und muss wöchentlich neu hergestellt werden.

### 4.6 Kalibrierstandards

- AFB2-Arbeitslösung I (100 mg/l)
  5 mg AFB2 werden in einen 50-ml-Messkolben eingewogen und 5 ml Methanol dazu pipettiert. Anschließend wird der Messkolben mit Acetonitril bis zur Markierung aufgefüllt und das AFB2 durch Umschütteln gelöst.

Die Arbeitslösung I wird in 10-ml-Braunglasflaschen mit Plastikschraubverschluss portioniert und bei −20 °C gelagert.

- AFB2-Arbeitslösung II (1 mg/l)
  10 μl der AFB2-Arbeitslösung I werden mit 990 μl Acetonitril versetzt und gemischt.

Die AFB2-Arbeitslösung II wird für jede Kalibrierung und zum Ansetzen der Qualitätskontrollproben neu hergestellt.

- OTA-Arbeitslösung (2,01 mg/l)
  40 μl der OTA-Stammlösung (10,05 mg/l Acetonitril) werden mit 160 μl Acetonitril versetzt und die Lösung gut gemischt.

Die OTA-Arbeitslösung wird für jede Kalibrierung und zum Ansetzen der Qualitätskontrollproben neu hergestellt.

- GT-Arbeitslösung I (500 mg/l)
  5 mg GT werden in einen 10-ml-Messkolben eingewogen und in ein wenig Acetonitril gelöst. Der Messkolben wird anschließend bis zur Markierung mit Acetonitril aufgefüllt.

Die GT-Arbeitslösung I wird in 1,5-ml-Polypropylengewindefläschchen portioniert und bei −20 °C gelagert.

- GT-Arbeitslösung II (100 mg/l)
  In 1,5-ml-Polypropylengewindefläschchen werden 200 μl der GT-Arbeitslösung I mit 800 μl Acetonitril versetzt und die Lösung wird gut gemischt.

Die GT-Arbeitslösung II wird für jede Kalibrierung und zum Ansetzen der Qualitätskontrollproben neu hergestellt.

- Dotierlösung 1 (DL 1)
  Die Dotierlösung 1 wird nach dem in Tabelle 1 gegebenen Pipettierschema in einem 1,5-ml-Polypropylengewindefläschchen angesetzt.

**Tab. 1:** Pipettierschema zur Herstellung der Dotierlösung 1

Analyt	**Lösung**	**Volumen** **[μl]**	**Acetonitril** **[μl]**	**Analytkonzentration** **[μg/l]**
AFB1	AFB1-Stammlösung	10	695	20
AFB2	AFB2-Arbeitslösung II	35		35
AFG1	AFG1-Stammlösung	10		20
AFG2	AFG2-Stammlösung	170		86,7
AFM1	AFM1-Stammlösung	70		35,4
OTA	OTA-Arbeitslösung	10		20,1

- Dotierlösung 2 (DL 2; 500 μg OTα/l)
  In einem 1,5-ml-Polypropylengewindefläschchen werden 50 μl der OTα-Stammlösung mit 950 μl Methanol gemischt.
- Dotierlösung 3 (DL 3; 1000 μg GT/l)
  In einem 1,5-ml-Polypropylengewindefläschchen werden 10 μl der GT-Arbeitslösung II mit 990 μl Acetonitril gemischt.

Die Dotierlösungen 1 bis 3 werden bei −20 °C gelagert und müssen wöchentlich neu hergestellt werden.

Die Kalibrierstandards werden in möglichst unbelastetem Urin angesetzt. Zur Herstellung der Kalibrierstandards werden die Dotierlösungen 1 bis 3 gemäß dem in Tabelle 2 gegebenen Pipettierschema zu 4 ml Urin pipettiert. In Tabelle 3 sind die Konzentrationen der Analyten in den jeweiligen Kalibrierstandards aufgeführt. Die Aufarbeitung der Kalibrierlösungen erfolgt analog zu den zu vermessenden Proben wie unter Abschnitt 5 angegeben, allerdings ohne Zugabe von ISTD.

**Tab. 2:** Pipettierschema zur Herstellung der Kalibrierstandards

Lösung	**DL 1** **[μl]**	**DL 2** **[μl]**	**DL 3** **[μl]**	**ISTD-DL** **[μl]**
DB^a)^	0	0	0	0
B^b)^	0	0	0	20
K1	2,5	8	6	20
K2	5	16	12	20
K3	10	24	18	20
K4	15	32	24	20
K5	20	40	30	20
K6	30	60	40	20

a) Doppelblindprobe

b) Blindprobe

**Tab.3:** Konzentration der Analyten und ISTDs in den Kalibrierstandards

Lösung	Konzentration [μg/l]									
	^13^C_17_-AFB1	^13^C_20_-OTA	AFB1	AFB2	AFG1	AFG2	AFM1	OTA	OTα	GT
DB^a)^	0	0	0	0	0	0	0	0	0	0
B^b)^	0,15	0,15	0	0	0	0	0	0	0	0
K1	0,15	0,15	0,013	0,022	0,013	0,054	0,022	0,013	1	1,5
K2	0,15	0,15	0,025	0,044	0,025	0,108	0,044	0,025	2	3
K3	0,15	0,15	0,05	0,088	0,05	0,217	0,088	0,05	3	4,5
K4	0,15	0,15	0,075	0,131	0,075	0,325	0,133	0,075	4	6
K5	0,15	0,15	0,1	0,175	0,1	0,434	0,177	0,101	5	7,5
K6	0,15	0,15	0,15	0,262	0,15	0,65	0,265	0,151	7,5	10

a) Doppelblindprobe

b) Blindprobe

### 4.7 Kontrollstandardlösung

Die Messung einer Kontrollstandardlösung wird zur Überprüfung des equilibrierten Messsystems eingesetzt (Über­prüfung von Druck, Peakintensitäten und Retentionszeiten). Der Kontrollstandard wird zu Beginn und am Ende einer Sequenz injiziert.

Zur Herstellung der Kontrollstandardlösung werden in einem 1,5-ml-Polypropylengewindefläschchen jeweils 20 μl der Dotierlösungen 1 bis 3 sowie 20 μl der ISTD-Dotierlösung mit 920 μl Gradientenlösung gemischt. Die Lösung wird bei −20 °C gelagert und wöchentlich frisch angesetzt. In Tabelle 4 sind die Konzentrationen der Analyten und ISTDs in der Kontrollstandardlösung aufgeführt.

**Tab.4:** Konzentrationen der Analyten und ISTDs in der Kontrollstandardlösung

Analyt/ISTD	**Konzentration** **[μg/l]**
AFB1	0,4
AFB2	0,7
AFG1	0,4
AFG2	1,7
AFM1	0,71
OTA	0,4
OTα	10
GT	20
^13^C_17_-AFB1	0,6
^13^C_20_-OTA	0,6

## 5 Probenahme, Probenaufbereitung und Festphasenextraktion

### 5.1 Probenahme

Die Urinproben werden in Polypropylen-Urinbechern gesammelt, aliquotiert und bis zur Probenaufbereitung bei −20 °C gelagert.

### 5.2 Probenaufbereitung

Vor der Probenaufbereitung werden die Urinproben auf Raumtemperatur gebracht und homogenisiert. Aus der Urin­probe werden 4 ml in ein 13-ml-Zentrifugenröhrchen mit rundem Boden gegeben, mit 4 ml hochreinem Wasser und 20 μl der ISTD-Dotierlösung versetzt und gut durchmischt. Anschließend werden die Urinproben bei 10 °C mit 2045 × *g* für 15 min zentrifugiert. Der Überstand wird in ein neues 13-ml-Zentrifugenröhrchen mit rundem Boden dekantiert.

### 5.3 Festphasenextraktion

Die Analyten werden mit Hilfe von Oasis-HLB-Kartuschen angereichert. Die Kartuschen werden mit 5 ml Methanol und anschließend mit 5 ml verdünnter Salzsäure (55 mM, pH = 1,3) konditioniert. Die Proben werden portionsweise ohne Anlegen eines Vakuums aufgegeben (Flussrate ca. 1 ml/min) und die 13-ml-Zentrifugenröhrchen mit 2 ml hochreinem Wasser gespült, das ebenfalls auf die Kartuschen gegeben wird. Nach Aufgabe der Proben wird die stationäre Phase jeweils mit 2 ml 2 %igem Methanol in hochreinem Wasser gewaschen. Die Kartuschen werden durch ein kurzes Anlegen von Vakuum (ca. 5 min) getrocknet. Bei der beschriebenen Konditionierung der Kartuschen und bei der Probenaufgabe dürfen die Kartuschen nicht trocken laufen.

Die Analyten werden mit 2 × 2,5 ml Methanol in ein 15-ml-Zentrifugenröhrchen mit konischem Boden, in dem 200 μl hochreines Wasser vorgelegt wurden, eluiert. Die Kartuschen werden unter leichtem Vakuum kurz trocken gesaugt. Anschließend werden die Eluate bei 40 °C unter Stickstoffstrom auf ca. 200 μl eingeengt. Die eingeengten Eluate werden mit 300 μl Gradientenlösung versetzt, auf einem Vortex-Schüttler homogenisiert und anschließend über Spritzen­vorsatzfilter in 1,5-ml-Gewindefläschchen aus Polypropylen filtriert.

## 6 Instrumentelle Arbeitsbedingungen

Die analytischen Messungen erfolgten an einer Gerätekonfiguration bestehend aus einem Flüssigkeitschromatographen (HPLC‑System: Nexera XR, Shimadzu Deutschland GmbH, Duisburg) und einem Tandem-Massenspektro­meter (AB SCIEX QTRAP 5500, AB SCIEX Germany GmbH, Darmstadt).

### 6.1 Hochleistungsflüssigkeitschromatographie

**Table:** 

HPLC‑Säule:	Kinetex^®^ Biphenyl; 2,6 μm; 100 × 2,1 mm
Vorsäule:	UHPLC‑Vorsäule Biphenyl; 2,1 mm ID
Temperatur Säulenofen:	40 °C
Temperatur Autosampler:	15 °C
Injektionsvolumen:	20 μl
Eluent A:	0,1 % Essigsäure und 1 mM Ammoniumacetat in hochreinem Wasser
Eluent B:	0,1 % Essigsäure und 1 mM Ammoniumacetat in Methanol
Gradientenprogramm:	siehe Tabelle 5

**Tab.5:** Gradientenprogramm für die Bestimmung von Aflatoxinen, Ochratoxin A, freiem Ochratoxin α und Gliotoxin in Urin

Zeit [min]	**Fluss** **[ml/min]**	**Eluent A** **[%]**	**Eluent B** **[%]**
0,01	0,45	98	2
2	0,45	98	2
5	0,45	20	80
5,2	0,45	2	98
8	0,45	2	98
8,01	0,45	98	2
11	0,45	98	2

### 6.2 Tandem-Massenspektrometrie

**Table:** 

Quelle:	TurboSpray
Ionisierungsmodus:	ESI, positiv bzw. negativ
Ionenspray-Spannung:	5500 V bzw. −4500 V
Quellentemperatur:	500 °C
Nebuliser-Gas:	Stickstoff, 80 psi
Turbo-Heater-Gas:	Stickstoff, 80 psi
Curtain-Gas:	Stickstoff, 35 psi
Kollisionsgas:	Stickstoff
Scan-Modus:	*Multiple Reaction Monitoring* (MRM)
Dwell-Time:	20 ms bzw. 60 ms
Parameterspezifische Einstellungen:	siehe Tabelle 6

Die gerätespezifischen Parameter müssen vom Anwender individuell für das eingesetzte MS/MS‑System ermittelt und eingestellt werden. Die in diesem Abschnitt genannten gerätespezifischen Parameter sind für das für die Methoden­entwicklung verwendete System bestimmt und optimiert worden.

Für die Analyten wurden jeweils zwei Massenübergänge ausgewählt. Ein Übergang dient zur Quantifizierung (Quantifier) und der andere zur Bestätigung (Qualifier). Für die ISTDs wurden zwei bzw. drei Massenübergänge verwendet. Die ausgewählten Übergänge sind zusammen mit den Retentionszeiten und weiteren MRM-Parametern in Tabelle 6 zusammengefasst. Die angegebenen Retentionszeiten dienen nur als Anhaltspunkt, der Anwender muss sich von der Trennleistung der von ihm verwendeten LC‑Säule und dem daraus resultierenden Retentionsverhalten der Substanzen überzeugen.

**Tab.6:** Retentionszeiten und MRM‑Parameter für die Bestimmung von Aflatoxinen, Ochratoxin A, freiem Ochratoxin α und Gliotoxin in Urin

Analyt/ISTD	**Retentionszeit** **[min]**	**Q1** **(*m/z*)**	**Q3** **(*m/z*)**	**DP** **[V]**	**EP** **[V]**	**CE** **[V]**	**CXP** **[V]**
AFB1	7,2	313	285^a)^	100	10	31	18
			241	100	10	49	18
AFB2	7,06	315	287,2^a)^	96	10	37	18
			259,2	96	10	43	18
AFG1	6,95	329	243^a)^	80	10	37	12
			200	80	10	53	12
AFG2	6,82	331	313,2	111	10	35	18
			245,2^a)^	111	10	43	14
AFM1	6,66	329,07	272,9^a)^	91	10	33	16
			228,9	91	10	55	14
OTA	6,72	404	239^a)^	91	10	32	12
			102	91	10	105	12
^13^C_20_-OTA	6,72	424,1	377,2^a)^	44	10	19	34
			203,1	60	10	59	12
			250,1	44	10	31	33
^13^C_17_-AFB1	7,2	330,3	301,2^a)^	80	10	35	16
			284,3	80	10	47	16
OTα	5,78	255	167^a)^	−55	−10	−34	−11
			211,1	−55	−10	−22	−15
GT	6,32	325,1	261^a)^	−35	−10	−14	−14
			243,2	−35	−10	−22	−17

a)  Quantifier

CE: Kollisionsenergie; CXP: Kollisionszellen-Austrittspotential; DP: Declustering-Potential; EP: Eingangspotential

## 7 Analytische Bestimmung

**Abb.2:**
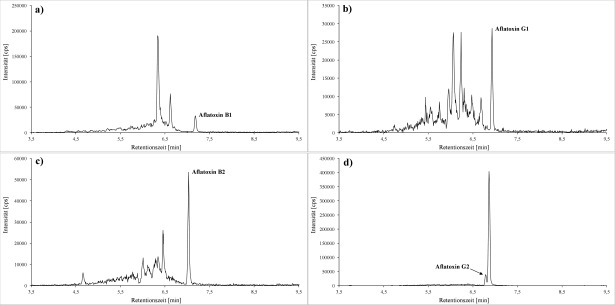
Quantifier-MRM‑Spuren für a) Aflatoxin B1 (0,013 μg/l; *m/z* 313 → 285), b) Aflatoxin G1 (0,013 μg/l; *m/z* 329 → 243), c) Aflatoxin B2 (0,022 μg/l; *m/z* 315 → 287,2) sowie d) Aflatoxin G2 (0,054 μg/l; *m/z* 331 → 245,2)

Von den nach Abschnitt 5 aufgearbeiteten Proben werden jeweils 20 μl in das LC‑MS/MS-System injiziert. Die Identi­fizierung der Analyten erfolgt anhand der spezifischen Ionenübergänge und Retentionszeiten. Die Abbildungen 2 und 3 zeigen beispielhaft Chromatogramme des K1-Kalibrierstandards.

**Abb.3:**
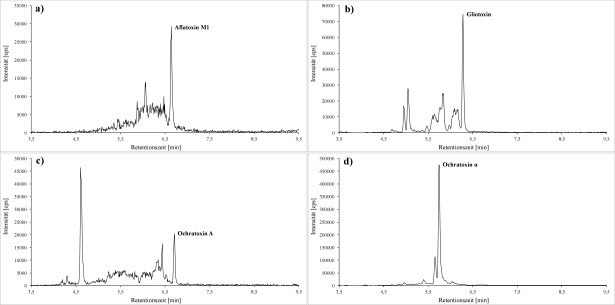
Quantifier-MRM‑Spuren für a) Aflatoxin M1 (0,022 μg/l; *m/z* 329 → 272,9), b) Gliotoxin (1,5 μg/l; *m/z* 325,1 → 261), c) Ochratoxin A (0,013 μg/l; *m/z* 404 → 239) sowie d) Ochratoxin α (1 μg/l; *m/z* 255 → 167)

## 8 Kalibrierung

Die unter Abschnitt 4.6 beschriebenen Kalibrierlösungen werden so wie die Proben aufgearbeitet (vgl. Abschnitt 5, ohne Zugabe von ISTD) und mittels LC‑MS/MS (vgl. Abschnitt 6) analysiert.

**Abb.4:**
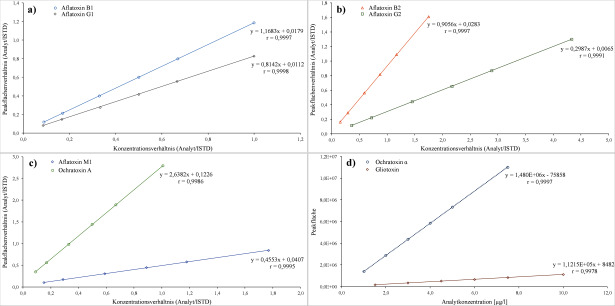
Kalibriergeraden für die Bestimmung von a) Aflatoxin B1 und Aflatoxin G1, b) Aflatoxin B2 und Aflatoxin G2, c) Aflatoxin M1 und Ochratoxin A sowie d) freiem Ochratoxin α und Gliotoxin in Urin (lineare Regression nach 1/x-Gewichtung)

Die Erstellung der Kalibriergeraden für die Aflatoxine und OTA erfolgt durch Auftragung der Quotienten aus der Peakfläche des Analyten und der Peakfläche des isotopenmarkierten ISTDs gegen den Quotienten der dotier­ten Konzentration des Analyten und der dotierten Konzentration des isotopenmarkierten ISTDs. Für die Quanti­fizierung der Aflatoxine und OTA werden die Peakflächen auf ^13^C_17_‑AFB1 bzw. ^13^C_20_‑OTA bezogen. Die Erstellung der Kalibriergeraden für OTα und GT erfolgt durch Auftragen der Peakflächen gegen die jeweils dotierte Analyt­konzentration. Für alle Analyten lag in den untersuchten Konzentrationsbereichen eine lineare Korrelation mit Korrelationskoeffizienten von r ≥ 0,995 vor (1/x-Gewichtung). Die Abbildung 4 zeigt beispielhaft die Kalibriergeraden für die Bestimmung der Aflatoxine sowie von OTA, OTα und GT in Urin.

## 9 Berechnung der Analysenergebnisse

Zur Berechnung der Gehalte an Aflatoxinen und OTA in einer Urinprobe wird der Quotient aus der Peakfläche des jeweiligen Analyten und der Peakfläche des ISTDs gebildet. Mithilfe der zur Analysenserie gehörigen Kalibrierfunktion kann aus dem ermittelten Quotienten der Analytgehalt in μg/l Urin berechnet werden. Für OTα und GT wird der Analytgehalt in μg/l Urin mithilfe der zur Analysenserie gehörigen Kalibrierfunktion aus der ermittelten Peakfläche berechnet.

## 10 Standardisierung der Messergebnisse und Qualitätssicherung

Zur Sicherung der Qualität der Analysenergebnisse wird gemäß den Richtlinien der Bundesärztekammer und den Angaben in dem von der Kommission veröffentlichten allgemeinen Kapitel verfahren (Bader et al. 2010; Bundes­ärztekammer 2014).

Im Rahmen der Qualitätssicherung werden mit jeder Kalibrierreihe in Urin angesetzte Blind- und Doppelblindproben gemessen. Die Doppelblindproben enthalten weder Analyt noch ISTD, die Blindproben werden mit den ISTDs dotiert, aber nicht mit den Analyten. Zudem wird in jeder Analysenserie ein Reagenzienleerwert (hochreines Wasser anstelle der Urinprobe) aufgearbeitet und gemessen.

Zur Präzisionskontrolle werden in jeder Analysenserie mindestens zwei Qualitätskontrollproben mit bekannter Konzentration der Analyten mituntersucht. Da käufliches Material nicht zur Verfügung steht, wird das Kontrollmaterial selbst hergestellt, indem Urin mit Standardlösungen der Analyten im relevanten Konzentrationsbereich dotiert wird (siehe Tabellen 7 und 8). Zusätzlich werden in jeder Sequenz die Kontrollstandardlösung (siehe Abschnitt 4.7) sowie undotierte Gradientenlösung mitgemessen und Spülschritte mit Methanol durchgeführt.

**Tab.7:** Pipettierschema zur Herstellung der Qualitätskontrollproben

Lösung	**DL 1** **[μl]**	**DL 2** **[μl]**	**DL 3** **[μl]**	**ISTD-DL** **[μl]**	**Urin** **[μl]**
Q_low_	7,5	20	15	20	4000
Q_high_	25	50	35	20	4000

**Tab.8:** Konzentration der Analyten und ISTDs in den Qualitätskontrollproben

Lösung	Konzentration [μg/l]									
	AFB1	AFB2	AFG1	AFG2	AFM1	OTA	OTα	GT	^13^C_17_-AFB1	^13^C_20_-OTA
Q_low_	0,0375	0,0654	0,0375	0,163	0,066	0,0377	2,5	3,75	0,15	0,15
Q_high_	0,13	0,22	0,13	0,54	0,22	0,13	6,25	8,75	0,15	0,15

## 11 Beurteilung des Verfahrens

Die Zuverlässigkeit des Verfahrens wurde durch eine umfassende Validierung sowie durch Nachstellung und Prüfung der Methode in einem zweiten, unabhängigen Labor bestätigt.

Von den Methodenprüfern wurden zusätzlich das Mykotoxin CIT und dessen Metabolit DH-CIT in die Methode integriert. Die Validierungsdaten für diese beiden Parameter sind im Anhang aufgeführt, allerdings wurden diese nicht unabhängig überprüft.

### 11.1 Präzision

Die Präzision in der Serie wurde bestimmt, indem die hergestellten Qualitätskontrollproben Q_low_ und Q_high_ sechsfach parallel aufgearbeitet und analysiert wurden. Die ermittelten Präzisionsdaten sind in Tabelle 9 dargestellt.

**Tab.9:** Präzision in der Serie für die Bestimmung der Aflatoxine sowie von Ochratoxin A, freiem Ochratoxin α und Gliotoxin in Urin

Analyt	**Dotierte Konzentration** **[μg/l]**	**Anzahl *n***	**Gemessene Konzentration** **[μg/l]**	Standardabweichung (rel.) ***s_w_*** **[%]**	Streubereich ***u*** **[%]**
AFB1	0,0375	6	0,0370	2,9	7,4
	0,13	6	0,1302	1,8	4,6
AFB2	0,0654	6	0,0683	2,4	6,2
	0,22	6	0,2239	2,2	5,6
AFG1	0,0375	6	0,0373	3,5	9,1
	0,13	6	0,1303	2,6	6,8
AFG2	0,163	6	0,1636	2,0	5,0
	0,54	6	0,5549	3,2	8,3
AFM1	0,066	6	0,0714	3,1	7,9
	0,22	6	0,2218	3,6	9,4
OTA	0,0377	6	0,0390	1,5	3,9
	0,13	6	0,1216	3,6	9,4
OTα	2,5	6	2,38	1,3	3,3
	6,25	6	5,84	4,0	10,4
GT	3,75	6	3,58	1,7	4,4
	8,75	6	8,13	4,9	12,7

Zur Bestimmung der Präzision von Tag zu Tag wurden die Kontrollmaterialien Q_low_ und Q_high_ je nach Analyt an sechs bis acht Tagen doppelt aufgearbeitet und analysiert. Die ermittelten Präzisionsdaten sind in Tabelle 10 dargestellt.

**Tab.10:** Präzision von Tag zu Tag für die Bestimmung der Aflatoxine sowie von Ochratoxin A, freiem Ochratoxin α und Gliotoxin in Urin

Analyt	**Dotierte Konzentration** **[μg/l]**	**Anzahl *n***	**Gemessene Konzentration** **[μg/l]**	Standardabweichung (rel.) ***s_w_*** **[%]**	Streubereich ***u*** **[%]**
AFB1	0,0375	6	0,0396	5,2	13,4
	0,13	8	0,1215	3,6	8,6
AFB2	0,0654	6	0,0673	2,2	5,6
	0,22	8	0,2106	3,6	8,5
AFG1	0,0375	6	0,0399	4,9	12,5
	0,13	8	0,1231	3,5	8,3
AFG2	0,163	6	0,1562	2,7	7,0
	0,54	8	0,5200	3,3	7,8
AFM1	0,066	6	0,0681	3,6	9,3
	0,22	8	0,2121	5,9	13,9
OTA	0,0377	6	0,0349	8,8	22,7
	0,13	7	0,1077	12,5	30,6
OTα	2,5	6	2,50	5,2	13,2
	6,25	8	6,22	6,5	15,3
GT	3,75	6	3,64	6,6	16,9
	8,75	8	9,08	8,6	20,4

### 11.2 Richtigkeit

Die Richtigkeit des Verfahrens wurde aus den Daten der Präzision in der Serie sowie der Präzision von Tag zu Tag ermit­telt. Die errechneten mittleren relativen Wiederfindungen für die einzelnen Analyten sind in Tabelle 11 aufgeführt.

**Tab.11:** Mittlere relative Wiederfindungen für die Bestimmung der Aflatoxine sowie von Ochratoxin A, freiem Ochratoxin α und Gliotoxin in Urin

Analyt	Dotierte Konzentration [μg/l]	Präzision in der Serie			Präzision von Tag zu Tag		
		Anzahl *n*	Wiederfindung (rel.) *r* [%]	Bereich [%]	Anzahl *n*	Wiederfindung (rel.) *r* [%]	Bereich [%]
AFB1	0,0375	6	98,8	95,4–103	6	106	98,8–113
	0,13	6	100	98,0–103	8	93,5	88,2–100
AFB2	0,0654	6	104	99,8–107	6	103	99,7–105
	0,22	6	102	98,3–104	8	95,7	91,8–102
AFG1	0,0375	6	99,4	94,8–104	6	107	99,4–112
	0,13	6	100	97,7–104	8	94,6	89,9–100
AFG2	0,163	6	100	97,4–103	6	95,8	92,6–100
	0,54	6	103	97,5–107	8	96,3	93,1–103
AFM1	0,066	6	108	105–114	6	103	98,1–108
	0,22	6	101	95,5–106	8	96,4	86,9–105
OTA	0,0377	6	103	102–105	6	92,5	84,0–103
	0,13	6	93,5	87,8–96,3	7	82,9	70,4–96,0
OTα	2,5	6	95,4	94,3–97,5	6	99,9	91,6–104
	6,25	6	93,4	90,3–100	8	99,5	93,4–112
GT	3,75	6	95,4	93,2–97,2	6	97,1	90,8–108
	8,75	6	92,9	86,8–97,1	8	104	92,9–117

### 11.3 Absolute Wiederfindung

Von den Prüfern der Methode wurden die aufarbeitungsbedingten Verluste bestimmt. Dazu wurden Urinproben vor der Aufarbeitung mit den Analyten auf drei Konzentrationsniveaus dotiert und die ISTDs zugegeben (Serie A) oder nach der Probenaufbereitung mit den maximal erwartbaren Mengen der Analyten versetzt (Serie B). Die Quotienten der Peakflächen der Probenserie A und der Probenserie B bilden die Analytverluste durch die Probenaufarbeitung ab und sind für die untersuchten Konzentrationen in Tabelle 12 zusammengestellt.

**Tab.12:** Absolute Wiederfindung für die Bestimmung der Aflatoxine sowie von Ochratoxin A, freiem Ochratoxin α und Gliotoxin in Urin (n = 6)

Analyt	**Dotierte Konzentration** [µg/l]	**Peakflächenverhältnis Serie A/Serie B**
AFB1	0,01	0,91
	0,0503	0,94
	0,1005	0,91
AFB2	0,0176	0,96
	0,0878	0,96
	0,1757	0,93
AFG1	0,01	0,87
	0,0508	0,92
	0,1015	0,86
AFG2	0,0426	0,97
	0,2129	0,94
	0,4259	0,90
AFM1	0,0175	0,91
	0,0877	0,95
	0,1754	0,91
OTA	0,01	0,90
	0,0502	0,95
	0,1003	0,90
OTα	0,77	0,85
	3,06	1,05
	5,1	1,02
GT	1,0	0,73
	4,5	0,83
	7,5	0,90

### 11.4 Nachweis- und Bestimmungsgrenzen

Die in Tabelle 13 aufgeführten Nachweisgrenzen wurden jeweils aus dem dreifachen und die Bestimmungsgrenzen aus dem zehnfachen Signal/Rausch-Verhältnis geschätzt. 

**Tab.13:** Nachweis- und Bestimmungsgrenzen für die Bestimmung der Aflatoxine sowie von Ochratoxin A, freiem Ochratoxin α und Gliotoxin in Urin

Analyt	**Nachweisgrenze** **[μg/l]**	**Bestimmungsgrenze** **[μg/l]**
AFB1	0,004	0,013
AFB2	0,007	0,022
AFG1	0,004	0,013
AFG2	0,02	0,054
AFM1	0,007	0,022
OTA	0,004	0,013
OTα	0,4	1,0
GT	0,5	1,5

### 11.5 Analytstabilität in der Urinmatrix

Die Analytstabilität in der Urinmatrix wurde bei Raumtemperatur, bei 4 °C und bei −20 °C untersucht. Die Stabilität bei Raumtemperatur wurde über einen Zeitraum von 24 Stunden untersucht und ist für die Probenvorbereitung relevant. Die Stabilität bei der Lagerung im Kühlschrank bei 4 °C wurde über einen Zeitraum von 48 Stunden untersucht und ist für eine Kurzzeitlagerung von Urinproben von Relevanz. Die Stabilität bei −20 °C ist für eine längere Probenlagerung wichtig und wurde nach einer Woche, nach zwei Wochen und nach viereinhalb Wochen bestimmt.

Zur Bestimmung der Analytstabilität wurden Q_low_- und Q_high_-Proben jeweils doppelt aufgearbeitet und analysiert. Als Akzeptanzkriterium wurde die Entscheidung 2002/657/EG der Europäischen Union zugrunde gelegt, welche eine Abweichung vom Nominalwert von −50 bis +20 % erlaubt (Europäische Kommission 2002).

Die Wiederfindungen der Analyten nach Lagerung bei Raumtemperatur, 4 °C und −20 °C lagen im Akzeptanzbereich, die Daten sind in Tabelle 14 aufgeführt.

**Tab.14:** Wiederfindung der Analyten nach Lagerung bei Raumtemperatur, 4 °C und −20 °C

Analyt	**Dotierte Konzentration [μg/l]**	**Raumtemperatur**	**4 °C**	**−20 °C**	**−20 °C**	**−20 °C**
		**Wiederfindung nach 24 h [%]**	**Wiederfindung nach 48 h [%]**	**Wiederfindung nach 1 Woche [%]**	**Wiederfindung nach 2 Wochen [%]**	**Wiederfindung nach 4,5 Wochen [%]**
AFB1	0,0375	107	110	101	90,6	91,8
	0,13	104	108	99,2	91,6	102
AFB2	0,0654	102	104	103	90,4	99,0
	0,22	99,6	102	97,8	91,3	110
AFG1	0,0375	103	112	83,6	72,6	110
	0,13	115	113	82,7	78,6	118
AFG2	0,163	95,8	98,0	92,6	88,2	98,2
	0,54	99,1	104	92,0	89,9	108
AFM1	0,066	96,8	103	86,9	84,1	118
	0,22	100	100	80,6	80,1	107
OTA	0,0377	82,7	88,8	106	102	91,4^a)^
	0,13	82,2	79,1	98,8	88,5	94,7^a)^
OTα	2,5	95,1	99,6	90,8	80,0	90,2
	6,25	88,9	81,2	87,4	92,8	110
GT	3,75	84,9	85,3	119	99,0	80,5^a)^
	8,75	92,8	91,8	113	112	83,2^a)^

a) Wiederfindung nach 5 Wochen

### 11.6 Analytstabilität in den messfertigen Proben

Die Stabilität der Analyten im Extrakt der aufgearbeiteten Qualitätskontrollproben wurde nach ein- sowie vierwöchiger Lagerung bei −20 °C bestimmt. Als Akzeptanzkriterium wurde wiederum die Entscheidung 2002/657/EG der Europäischen Union zugrunde gelegt (Europäische Kommission 2002). Die Ergebnisse zur Analytstabilität in den messfertigen Proben sind in Tabelle 15 zusammengestellt. Die Wiederfindungen der Analyten in den Extrakten lagen nach Lagerung bei −20 °C zwischen 80,8 % und 105 %.

**Tab.15:** Analytstabilität in den messfertigen Proben nach Lagerung bei −20 °C

Analyt	**Dotierte Konzentration** **[μg/l]**	**Wiederfindung nach 1 Woche** **[%]**	**Wiederfindung nach 4 Wochen** **[%]**
AFB1	0,0375	105	97,4
	0,13	104	100
AFB2	0,0654	102	101
	0,22	104	104
AFG1	0,0375	82,3	108
	0,13	80,8	105
AFG2	0,163	96,6	108
	0,54	98,8	104
AFM1	0,066	94,5	114
	0,22	88,2	111
OTA	0,0377	105	101^a)^
	0,13	98,4	106^a)^
OTα	2,5	95,0	96,0
	6,25	90,1	110
GT	3,75	102	105^a)^
	8,75	109	105^a)^

a) Wiederfindung nach 2 Wochen

### 11.7 Störeinflüsse

Für die Herstellung der Kalibrierstandards wurden Urine verschiedener Personen auf die Gehalte der zu messenden Analyten getestet. Da in fast allen untersuchten Urinproben nicht vernachlässigbare Gehalte an OTA zu finden waren, konnte kein Poolurin verwendet werden. Die Kalibrierstandards wurden schließlich in einem nahezu unbelasteten nativen Urin angesetzt, der in größeren Mengen gesammelt und bei −20 °C gelagert wurde.

## 12 Diskussion der Methode

Diese Methode ermöglicht die zuverlässige Bestimmung von Aflatoxinen, OTA, freiem OTα und GT in Urin. Die Vali­dierungsdaten zeigen eine gute Reproduzierbarkeit, Richtigkeit und Empfindlichkeit der Methode. Die von den Ent­wicklern der Methode ermittelte Bestimmungsgrenze für OTA ist ähnlich wie die anderer Methoden (Föllmann et al. 2016). Für OTα lag die Bestimmungsgrenze mit 1 μg/l Urin über den in anderen Studien angegebenen Werten (Njumbe Ediage et al. 2012). Für GT liegen bislang keine Daten aus anderen Studien vor. Die bei der Methodenentwicklung für die Aflatoxine ermittelten Bestimmungsgrenzen entsprachen zum Teil denen anderer Methoden (Gerding et al. 2015; Schmidt et al. 2021), lagen teilweise aber auch unterhalb der in anderen Studien angegeben Werte (Penczynski et al. 2022; Schmidt et al. 2021). Bei der externen Methodenprüfung wurden für alle Analyten noch niedrigere Bestim­mungsgrenzen ermittelt, was wahrscheinlich auf die Verwendung eines empfindlicheren Tandem-Massen­spektro­meters zurückzuführen ist.

Im Rahmen der Methodenentwicklung wurden verschiedene SPE‑Materialien zur Aufreinigung und Anreicherung sowie verschiedene HPLC‑Trennsäulen und Eluentengemische für die HPLC getestet. Was die analytische Säule anbe­langt, so war die Kinetex Biphenyl-Säule (Phenomenex) die einzige, die zu einer befriedigenden Auftrennung der Mykotoxine führte. Mit anderen RP-Säulen war es zudem nicht möglich, die Analyten von der Urinmatrix abzutrennen. 

Die Auswertung der Aflatoxine erfolgte mit dem ISTD ^13^C_17_‑AFB1. Zu Beginn der Methodenentwicklung wurden weitere ISTDs (^13^C_17_‑AFB2 und ^13^C_17_‑AFM1) verwendet. Umfangreiche Prüfungen ergaben jedoch, dass der ISTD ^13^C_17_‑AFB1 für alle untersuchten Aflatoxine eingesetzt werden kann. Die Auswertung von OTA erfolgte mit ^13^C_20_‑OTA, die von OTα und GT ohne ISTD.

Die Konzentrationen der Kalibrierstandards wurden während der Methodenentwicklung an Realproben einer laufenden Studie angepasst, konkret wurden die Kalibrierbereiche der einzelnen Analyten nach unten korrigiert. Die Konzentrationen der ISTDs wurden allerdings nicht geändert, so dass Anwender der Methode diese gegebenenfalls in geringeren Konzentrationen einsetzen können.

Die Untersuchung der Analytstabilität in der Urinmatrix sowie in den messfertigen Proben bestätigte die bereits in der Literatur beschriebene Instabilität von GT in Urin (Cerqueira et al. 2014), so dass die Lösungen für GT wöchentlich neu angesetzt werden sollten. 

Mit dieser Methode untersuchten die Prüfer der Methode 32 Urinproben (24-h-Urine) von 16 Rohköstlern sowie 16 Kontrollpersonen (Veganer und Omnivoren). In den meisten Proben wurden quantifizierbare Gehalte an OTA (94 %) und CIT (94 %) detektiert. Im Vergleich zur Kontrollgruppe wurden im Urin der Rohköstler geringere Mengen OTA und CIT gefunden. Aflatoxine, freies OTα oder GT wurden in keiner der Proben nachgewiesen.

**Verwendete Messgeräte** HPLC‑Anlage mit binärer Pumpe, Autosampler, Säulenofen und Degasser (Nexera XR, Shimadzu Deutschland GmbH, Duisburg); Triple-Quadrupol-Massenspektrometer (Modell AB SCIEX QTRAP 5500 mit Elektrosprayionisierung, AB SCIEX Germany GmbH, Darmstadt)

## Figures and Tables

**Tab.16:** Pipettierschema zur Herstellung der Kalibrierlösungen für die Bestimmung von Citrinin und Dihydrocitrinon in Urin

Lösung	CIT- und DH-CIT-DL [μl]	CIT-Konzentration [µg/l]	DH-CIT-Konzentration [µg/l]	^13^C_13_-CIT-DL [μl]	^13^C_13_-CIT-Konzentration [μg/l]
DB^a)^	0	0	0	0	0
B^b)^	0	0	0	20	1,017
K1	4^c)^	0,001	0,001	20	1,017
K2	10^c)^	0,0025	0,0025	20	1,017
K3	20^c)^	0,005	0,005	20	1,017
K4	30^c)^	0,0075	0,0075	20	1,017
K5	40^c)^	0,01	0,01	20	1,017
K6	100^c)^	0,025	0,025	20	1,017
K7	4	0,05	0,05	20	1,017
K8	8	0,1	0,1	20	1,017
K9	40	0,5	0,5	20	1,017
K10	80	1,001	1	20	1,017
K11	160	2,002	2	20	1,017

a) Doppelblindprobe

b) Blindprobe

c) vorheriger Verdünnungsschritt: 20 μl der Dotierlösung mit 980 μl Acetonitril

**Tab.17:** Konzentrationen der Analyten und des ISTDs in der Kontrollstandardlösung

Analyt/ISTD	**Konzentration** **[μg/l]**
CIT	1,001
DH-CIT	1,0
^13^C_13_-CIT	2,034

**Table:** 

HPLC‑Säule:	Kinetex® Biphenyl; 2,6 μm; 100 × 2,1 mm
Vorsäule:	UHPLC‑Vorsäule Biphenyl; 2,1 mm ID
Temperatur Säulenofen:	40 °C
Temperatur Autosampler:	10 °C
Injektionsvolumen:	10 μl
Eluent A:	0,1 % Essigsäure und 1 mM Ammoniumacetat in hochreinem Wasser
Eluent B:	0,1 % Essigsäure und 1 mM Ammoniumacetat in Methanol
Gradientenprogramm:	siehe Tabelle 18

**Tab.18:** Gradientenprogramm für die Bestimmung von Citrinin und Dihydrocitrinon in Urin

Zeit [min]	**Fluss** **[ml/min]**	**Eluent A** **[%]**	**Eluent B** **[%]**
0	0,45	95	5
1	0,45	95	5
8	0,45	2	98
9	0,45	2	98
9,01	0,45	95	5
11	0,45	95	5

**Table:** 

Quelle:	TurboSpray
Ionisierungsmodus:	ESI negativ
Ionenspray-Spannung:	−4500 V
Quellentemperatur:	450 °C
Nebuliser-Gas:	Stickstoff, 60 psi
Turbo-Heater-Gas:	Stickstoff, 50 psi
Curtain-Gas:	Stickstoff, 40 psi
Kollisionsgas:	Stickstoff
Scan-Modus:	*Multiple Reaction Monitoring* (MRM)
Parameterspezifische Einstellungen:	siehe Tabelle 19

**Tab.19:** Retentionszeiten und MRM‑Parameter für die Bestimmung von Citrinin und Dihydrocitrinon in Urin

Analyt/ISTD	Retentionszeit [min]	Q1 (*m/z*)	Q3 (*m/z*)	Dwell-time [msec]	DP [V]	CE [V]	CXP [V]
CIT	5,23	280,9 [M-H+CH3OH]–	248,9^a)^	40	−60	−22	−15
			204,9	40	−60	−32	−11
		248,9 [M-H]–	176,9	40	−150	−30	−11
DH-CIT	5,13	264,9 [M-H]–	220,9^a)^	40	−50	−30	−13
			246,9	40	−50	−30	−15
			176,9	40	−50	−34	−17
^13^C_13_-CIT	5,23	293,99 [M-H+CH3OH]–	261,96^a)^	40	−40	−24	−7
			217,0	40	−40	−32	−21

a) Quantifier

CE: Kollisionsenergie; CXP: Kollisionszellen-Austrittspotential; DP: Declustering-Potential

**Abb.5:**
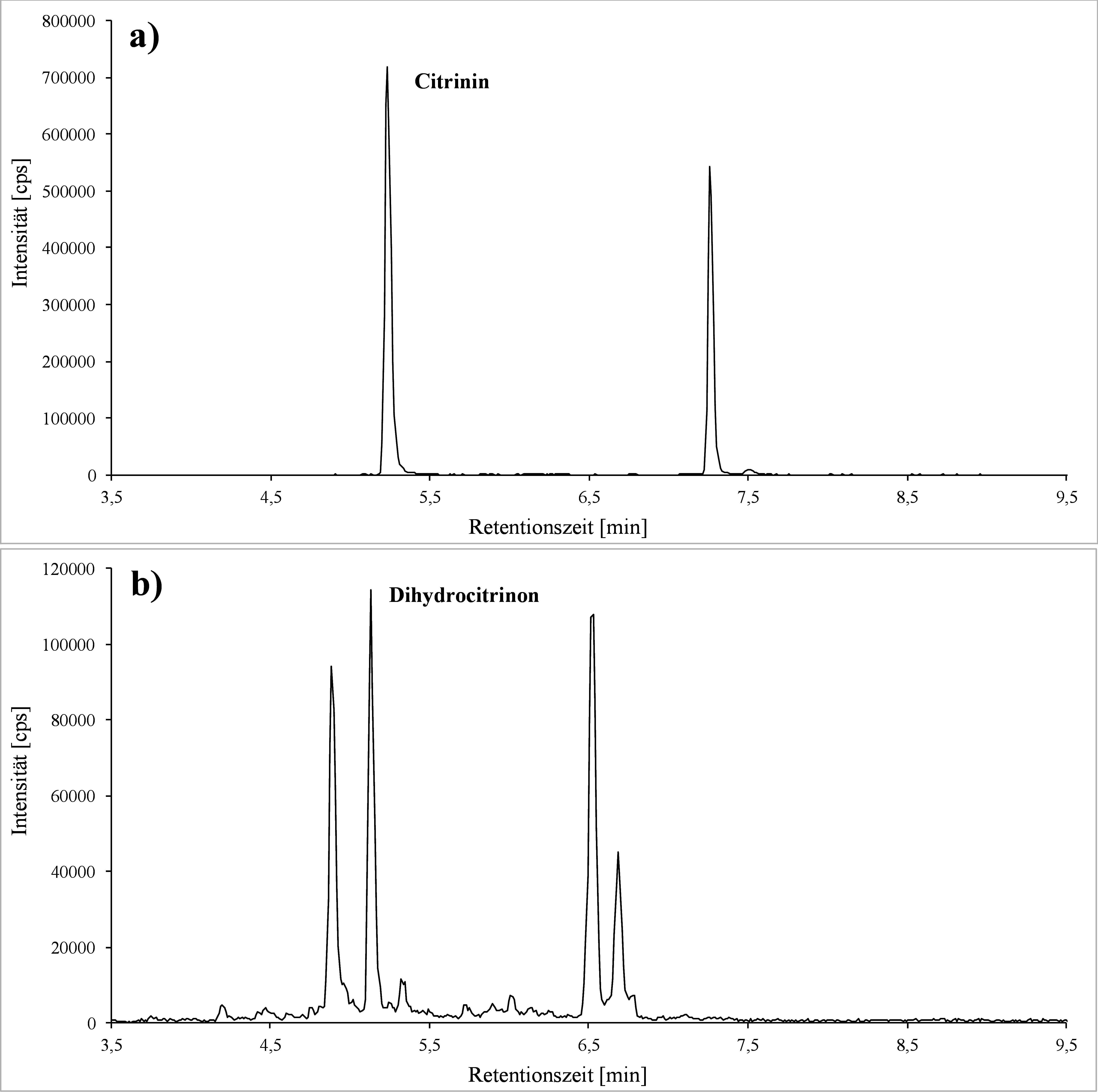
Chromatogramme von Leerurin dotiert mit a) 0,5 μg Citrinin/l (Quantifier *m/z* 281 → 249) sowie mit b) 0,5 μg Dihydrocitrinon/l (Quantifier *m/z* 265 → 221)

**Abb.6:**
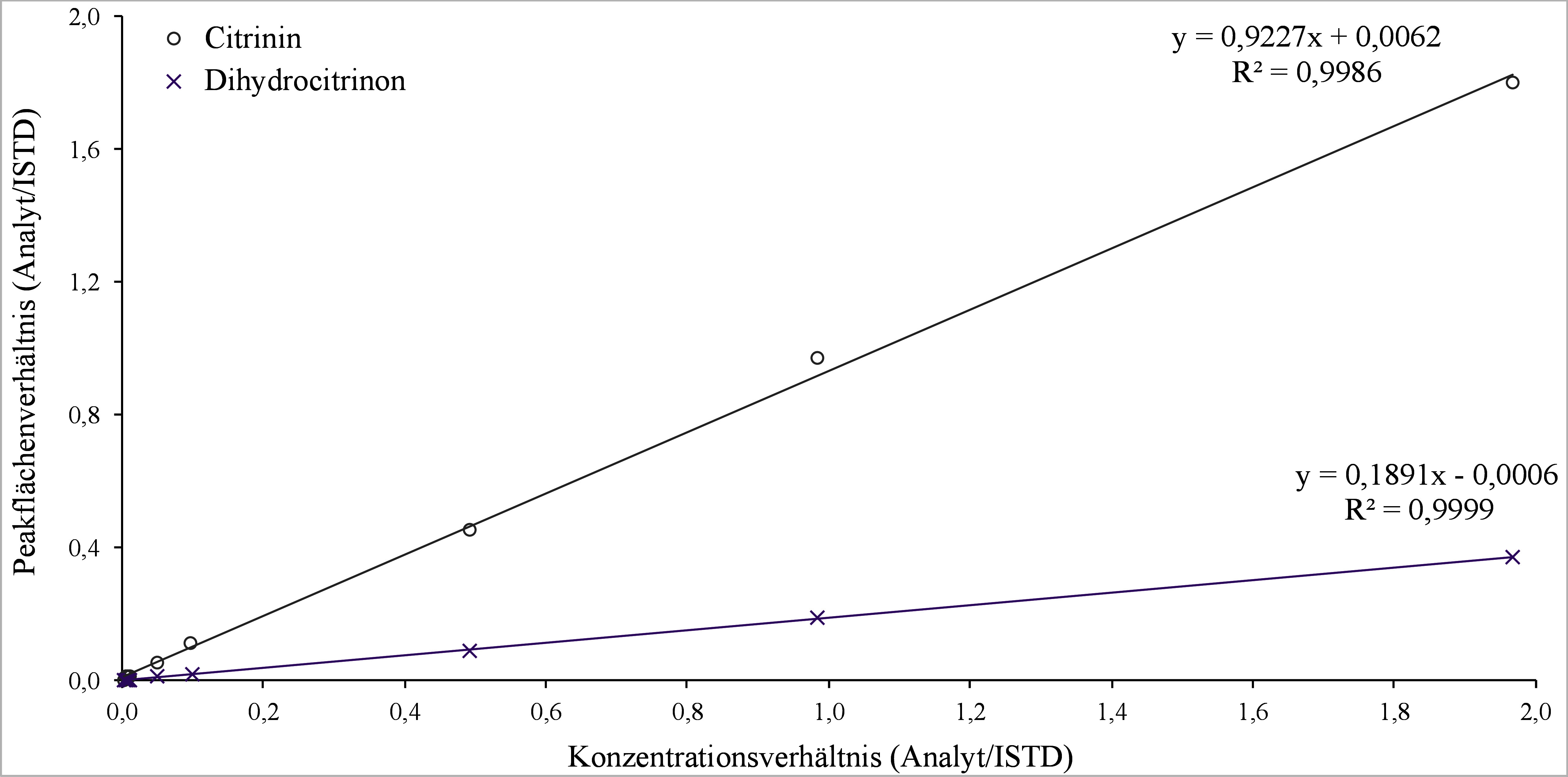
Kalibriergerade für die Bestimmung von Citrinin und Dihydrocitrinon (lineare Regression ohne Gewichtung)

**Tab.20:** Präzision in der Serie für die Bestimmung von Citrinin und Dihydrocitrinon in Urin

Analyt	**Dotierte Konzentration** **[μg/l]**	**Anzahl *n***	**Gemessene Konzentration** **[μg/l]**	**Standardabweichung (rel.) *s_w_*** **[%]**	Streubereich ***u*** **[%]**
CIT	0,01	6	0,01	3,1	9,4
	0,1	6	0,09	6,0	16,5
	1,0	6	0,93	6,5	17,2
DH‑CIT	0,01	6	0,0108	14,0	37,5
	0,1	6	0,08	3,3	10,1
	1,0	6	0,89	8,9	23,9

**Tab.21:** Präzision von Tag zu Tag für die Bestimmung von Citrinin und Dihydrocitrinon in Urin

Analyt	**Dotierte Konzentration** **[μg/l]**	**Anzahl *n***	**Gemessene Konzentration** **[μg/l]**	**Standardabweichung (rel.) *s_w_*** **[%]**	Streubereich ***u*** **[%]**
CIT	0,01	6	0,01	6,4	17,5
	0,1	6	0,10	5,9	15,2
	1,0	6	0,98	2,1	6,2
DH‑CIT	0,01	6	0,01	19,3	54,7
	0,1	6	0,09	5,4	12,1
	1,0	6	0,96	3,4	9,9

**Tab.22:** Mittlere relative Wiederfindungen für die Bestimmung von Citrinin und Dihydrocitrinon in Urin

Analyt	Dotierte Konzentration [μg/l]	Präzision in der Serie			Präzision von Tag zu Tag		
		Anzahl *n*	Wiederfindung (rel.) *r* [%]	Bereich [%]	Anzahl *n*	Wiederfindung (rel.) *r* [%]	Bereich [%]
CIT	0,01	6	94,1	89,3–98,2	6	99,8	90,4–108
	0,1	6	88,6	79,7–94,3	6	99,0	91,2–106
	1,0	6	93,3	89,1–105	6	98,5	89,1–105
DH‑CIT	0,01	6	108	93,0–134	6	108	75,9–135
	0,1	6	80,9	77,0–85,2	6	88,4	82,3–93,1
	1,0	6	88,5	81,2–102	6	96,4	90,7–100

**Tab.23:** Absolute Wiederfindungen für die Bestimmung von Citrinin und Dihydrocitrinon in Urin (n = 6)

Analyt	**Dotierte Konzentration** **[µg/l]**	**Peakflächenverhältnis Serie A/Serie B**
CIT	0,01	1,03
	0,1	0,93
	1,0	0,99
DH-CIT	0,01	1,04
	0,1	0,95
	1,0	1,01

**Tab.24:** Nachweis- und Bestimmungsgrenzen für die Bestimmung von Citrinin und Dihydrocitrinon in Urin

Analyt	**Blindurin**		**Poolurin**	
	**Nachweisgrenze** **[μg/l]**	**Bestimmungsgrenze** **[μg/l]**	**Nachweisgrenze** **[μg/l]**	**Bestimmungsgrenze** **[μg/l]**
CIT	0,0003^a)^	0,001^a)^	0,01^b)^	0,025^b)^
DH-CIT	0,0075	0,01	0,5^b)^	0,5^b)^

a) Der Blindurin enthielt niedrige Hintergrundgehalte an Citrinin. Zur Abschätzung von Nachweis- und Bestimmungsgrenze wurden die S/N-Werte aller Proben (Abszisse) gegen die Nominalkonzentrationen (Ordinate) aufgetragen. Nach Abzug des mittleren S/N-Wertes aus undotierten Blindurinproben, wurden die Nachweis- und Bestimmungsgrenzen aus den resultierenden Regressionsfunktionen abgeschätzt und visuell bestätigt.

b) Der Poolurin enthielt niedrige Grundwerte von CIT und DH-CIT. Zur Abschätzung von Nachweis- und Bestimmungsgrenze wurden die S/N-Werte aller Proben (Abszisse) gegen die Nominalkonzentrationen (Ordinate) aufgetragen. Nach Abzug des mittleren S/N-Wertes aus undotierten Poolurinproben, wurden Nachweis- und Bestimmungsgrenze aus den resultierenden Regressionsfunktionen abgeschätzt und visuell bestätigt.

## Anhang

Bei der Methodenprüfung wurden – zusätzlich zu den Aflatoxinen, OTA, freiem OTα und GT – CIT und dessen Metabolit DH-CIT in die Methode integriert. Die allgemeine Vorgehensweise entsprach dabei der beschriebenen Methode der Entwickler, so dass an dieser Stelle nur die zur Quantifizierung von CIT und DH-CIT benötigten Infor­mationen ergänzend dargestellt werden. Die von den Prüfern der Methode erhobenen Validierungsdaten sind im Folgenden aufgeführt, allerdings wurden diese nicht unabhängig überprüft.

### Geräte, Chemikalien und Lösungen

#### Geräte

- UPLC-Anlage mit binärer Pumpe und Säulenofen (z. B. Acquity UPLC I-Class mit SM‑FTN-Sample Manager, Waters GmbH, Eschborn)
- Tandem-Massenspektrometer (z. B. AB SCIEX QTRAP 6500, AB SCIEX Germany GmbH, Darmstadt)

#### Referenzstandards und ISTD

- Citrinin, 100,1 µg/ml in Acetonitril (z. B. Romer Labs Division Holding GmbH, Getzersdorf, Österreich)
- Dihydrocitrinon (z. B. AnalytiCon Discovery GmbH, Potsdam)
- ^13^C_13_‑Citrinin, 10,17 µg/ml in Acetonitril (z. B. Romer Labs Division Holding GmbH, Getzersdorf, Österreich)

#### Lösungen – Referenzstandards und ISTD

- ^13^C_13_-CIT-Dotierlösung (^13^C_13_-CIT-DL; 203,4 μg ^13^C_13_-CIT/l)
  In einem 1,5-ml-Polypropylengewindefläschchen werden 20 μl des ^13^C_13_-CIT-Standards mit 980 μl Acetonitril gemischt.

Die ^13^C_13_-CIT-Dotierlösung wurde bei −80 °C gelagert. Bei dieser Lagerungstemperatur war der Analyt über einen Zeitraum von zwölf Wochen stabil.

- CIT-Arbeitslösung (1,001 mg/l)
  100 μl der CIT-Stammlösung werden in einen 10 ml-Messkolben pipettiert. Der Messkolben wird anschließend bis zur Markierung mit Acetonitril aufgefüllt.

Die CIT-Arbeitslösung wird bei −80 °C gelagert und ist drei Monate stabil.

- DH-CIT-Arbeitslösung I (500 mg/l)
  5 mg DH-CIT werden in einen 10-ml-Messkolben eingewogen und in ein wenig Acetonitril gelöst. Der Messkolben wird anschließend bis zur Markierung mit Acetonitril aufgefüllt.

Die DH-CIT-Arbeitslösung I wird bei −80 °C gelagert und ist drei Monate stabil.

- DH-CIT-Arbeitslösung II (5 mg/l)
  100 μl der DH-CIT-Arbeitslösung I werden in einen 10-ml-Messkolben pipettiert. Der Messkolben wird anschließ­end bis zur Markierung mit Acetonitril aufgefüllt.

Die DH-CIT-Arbeitslösung II wird für jede Kalibrierung und zum Ansetzen der Qualitätskontrollproben neu hergestellt.

- CIT- und DH-CIT-Dotierlösung (CIT- und DH-CIT-DL; 50,05 μg CIT/l und 50,0 μg DH-CIT/l)
  In einem 1,5-ml-Polypropylengewindefläschchen werden 50 μl der CIT-Arbeitslösung und 10 μl der DH-CIT-Arbeitslösung II mit 940 μl Acetonitril gemischt.

Die CIT- und DH-CIT-Dotierlösung wird für jede Kalibrierung und zum Ansetzen der Qualitätskontrollproben neu hergestellt.

#### Kalibrierstandards

Bei der Methodenprüfung wurden die Kalibrierlösungen in nahezu unbelastetem nativem Urin angesetzt. Da Urin­proben normalerweise messbare Gehalte verschiedener Mykotoxine aufweisen, wurde Urin von einer Person gesam­melt, die fünf Tage auf Getreideprodukte, Reis, Milchprodukte, Gewürze, Wein sowie Kaffee verzichtet hatte. Die Mykotoxingehalte dieses Urins lagen in der Regel unter den Nachweisgrenzen.

Zur Herstellung der Kalibrierstandards pipettierten die Methodenprüfer die Dotierlösungen gemäß dem in Tabelle 16 angegebenen Pipettierschema zu 4 ml Urin. Für die Analytkonzentrationen im Bereich von 0,001 μg/l bis 0,025 μg/l wurde die CIT- und DH-CIT-Dotierlösung weiter verdünnt.

Die Aufarbeitung der Kalibrierlösungen erfolgt analog zu den Proben wie unter Abschnitt 5 angegeben, allerdings ohne Zugabe von ISTD.

Die Kalibrierstandards sind dafür ausgelegt, den linearen Bereich der Detektion sowie die Nachweis- und Bestim­mungsgrenzen zu bestimmen. Zur Kalibrierung werden alle elf Lösungen verwendet, wobei die Konzentrationen unterhalb der Bestimmungsgrenze der jeweiligen Analyten nicht in die Kalibrierung eingeschlossen werden.

#### Kontrollstandardlösung

Die Kontrollstandardlösung wurde durch Verdünnen der CIT- und DH-CIT-Dotierlösung mit Gradientenlösung hergestellt, in 250-μl-Volumina aliquotiert und bei −80 °C gelagert. In Tabelle 17 sind die CIT und DH-CIT-Konzentrationen in dieser Lösung aufgeführt.

### Analytische Bestimmung

Von den aufgearbeiteten Proben werden jeweils 10 μl in das LC‑MS/MS-System injiziert. Abbildung 5 zeigt beispielhaft Chromatogramme für CIT und DH-CIT.

### Kalibrierung

Für die Erstellung der Kalibriergeraden zur Bestimmung von CIT und DH-CIT werden die Peakflächen jeweils auf ^13^C_13_-CIT bezogen und gegen die Quotienten der dotierten Konzentration des Analyten und der dotierten Konzentra­tion des ISTDs aufgetragen. Für CIT und DH-CIT lag in den untersuchten Konzentrationsbereichen eine lineare Korrelation mit Bestimmtheitsmaßen von R^2^ ≥ 0,998 vor (lineare Regression ohne Gewichtung). In Abbildung 6 sind beispielhaft die Kalibriergeraden für die Bestimmung von CIT und DH-CIT in Urin dargestellt.

### Berechnung der Analysenergebnisse

Zur Berechnung des CIT- oder DH-CIT-Gehaltes einer Probe wird jeweils der Quotient aus der Peakfläche des Analyten und der Peakfläche des ^13^C_13_-CITs gebildet. Mithilfe der zur Analysenserie gehörigen Kalibrierfunktion kann aus den ermittelten Quotienten der Analytgehalt in μg/l Urin berechnet werden.

### Standardisierung der Messergebnisse und Qualitätssicherung

In den drei bei der Methodenprüfung verwendeten Kontrollmaterialien lagen die Konzentrationen für CIT und DH-CIT bei 0,01 μg/l, 0,1 μg/l sowie 1 μg/l.

### Beurteilung des Verfahrens

Die Validierungsdaten für CIT und DH-CIT wurden nicht unabhängig überprüft.

#### Präzision

Für CIT und DH-CIT wurde die Präzision in der Serie von den Prüfern der Methode bestimmt, indem die drei Kontroll­materialien jeweils sechsfach aufgearbeitet und analysiert wurden.

Für die Präzision von Tag zu Tag wurden die drei Kontrollmaterialien an sechs Tagen aufgearbeitet und analysiert.

#### Richtigkeit

Die Richtigkeit des Verfahrens wurde aus den Daten der Präzision in der Serie sowie der Präzision von Tag zu Tag ermittelt.

#### Absolute Wiederfindung

Auch für CIT und DH-CIT wurden die aufarbeitungsbedingten Verluste bestimmt. Dazu wurden Urinproben vor der Aufarbeitung mit den Analyten sowie den ISTDs dotiert (Serie A) oder nach der Probenaufbereitung mit den maximal erwartbaren Mengen der Analyten versetzt (Serie B). Die Quotienten der Peakflächen der Probenserie A und der Probenserie B bilden den Analytverlust durch die Probenaufarbeitung ab.

#### Nachweis- und Bestimmungsgrenzen

Die Nachweis- und Bestimmungsgrenzen für die Bestimmung von CIT und DH-CIT wurden aus dem dreifachen bzw. zehnfachen Signal/Rausch-Verhältnis berechnet.

Der sehr matrixarme Leerurin führte in Kombination mit einem sensitiveren Tandem-Massenspektrometer (AB SCIEX QTRAP 6500 statt AB SCIEX QTRAP 5500) zu sehr niedrigen Nachweis- und Bestimmungsgrenzen. In einer aus fünf Individualurinen gepoolten Urinprobe fielen die Nachweis- und Bestimmungsgrenzen höher aus (Tabelle 24).

### Störeinflüsse und Diskussion

Mit der beschriebenen Methode lassen sich CIT und DH-CIT sensitiv und zuverlässig in Humanurin bestimmen. Die unter Verwendung von gepooltem Urin erhobenen Bestimmungsgrenzen von 0,025 µg CIT pro Liter Urin und 0,5 µg DH-CIT pro Liter Urin erlauben die Quantifizierung der ernährungsbedingten Hintergrundbelastung in der Allgemeinbevölkerung.

Die Validierungsdaten für CIT und DH-CIT zeigen eine gute Reproduzierbarkeit, Richtigkeit und Empfindlichkeit der Methode. So liegen die Standardabweichungen sowohl bei der Präzision in Serie als auch bei der Präzision von Tag zu Tag – für alle Analytkonzentrationen oberhalb der jeweiligen Bestimmungsgrenze – unter 10 %. Standard­abweichungen von 14,0 % und 19,3 % ergaben sich für den in Höhe der Bestimmungsgrenze dotierten DH-CIT-Standard (0,01 µg DH-CIT pro Liter).

Die aus den Präzisionsdaten berechneten mittleren relativen Wiederfindungen lagen zwischen 80,9 % und 108 % und können damit als sehr gut bezeichnet werden. Darüber hinaus zeigt die absolute Wiederfindung, dass es durch die Probenaufarbeitung zu keinen aufarbeitungsbedingten Analytverlusten kommt.

**Verwendete Messgeräte** UPLC-Anlage mit binärer Pumpe und Säulenofen (z. B. Acquity UPLC I-Class mit SM‑FTN-Sample Manager, Waters GmbH, Eschborn); Tandem-Massenspektrometer (z. B. AB SCIEX QTRAP 6500, AB SCIEX Germany GmbH, Darmstadt)

## References

[id_DUK_380] Al-Jaal Belqes Ahmad, Jaganjac Morana, Barcaru Andrei, Horvatovich Peter, Latiff Aishah (2019). Aflatoxin, fumonisin, ochratoxin, zearalenone and deoxynivalenol biomarkers in human biological fluids: a systematic literature review, 2001–2018. Food Chem Toxicol.

[id_DUK_381] Bader M., Barr D., Göen T., Schaller K. H., Scherer G., Angerer J., Angerer J, Hartwig A (2010). Analytische Methoden zur Prüfung gesundheitsschädlicher Arbeitsstoffe.

[id_DUK_382] Berger Marion, Marske Lennart, Monien Bernhard, Siodlaczek Solveigh, Göen T., Hartwig A., MAK Commission (2025). Mykotoxine – Bestimmung von Deoxynivalenol und Deepoxydeoxynivalenol in Urin mittels LC-MS/MS. Biomonitoring-Methode. MAK Collect Occup Health Saf.

[id_DUK_383] Boonen Jente, Malysheva Svetlana V., Taevernier Lien, Diana Di Mavungu José, De Saeger Sarah, De Spiegeleer Bart (2012). Human skin penetration of selected model mycotoxins. Toxicology.

[id_DUK_384] Brera C., Caputi R., Miraglia M., Iavicoli I., Salerno A., Carelli G. (2002). Exposure assessment to mycotoxins in workplaces: aflatoxins and ochratoxin A occurrence in airborne dusts and human sera. Microchem J.

[id_DUK_385] Bundesärztekammer (2014). Richtlinie der Bundesärztekammer zur Qualitätssicherung laboratoriumsmedizinischer Untersuchungen. Dtsch Ärztebl.

[id_DUK_386] Cerqueira Letícia B., de Francisco Thais M. G., Gasparetto João C., Campos Francinete R., Pontarolo Roberto (2014). Development and validation of an HPLC-MS/MS method for the early diagnosis of aspergillosis. PLoS ONE.

[id_DUK_387] EFSA Panel on Contaminants in the Food ChainCONTAM (2012). Scientific opinion on the risks for public and animal health related to the presence of citrinin in food and feed. EFSA J.

[id_DUK_388] Schrenk Dieter, Bignami Margherita, Bodin Laurent, Chipman James Kevin, Mazo Jesús, Grasl‐Kraupp Bettina, Hogstrand Christer, Hoogenboom Laurentius Ron, Leblanc Jean‐Charles, Nebbia Carlo Stefano, Nielsen Elsa, Ntzani Evangelia, Petersen Annette, Sand Salomon, Schwerdtle Tanja, Vleminckx Christiane, Marko Doris, Oswald Isabelle P, Piersma Aldert, Routledge Michael, Schlatter Josef, Baert Katleen, Gergelova Petra, Wallace Heather, EFSA Panel on Contaminants in the Food ChainCONTAM (2020). Risk assessment of aflatoxins in food. EFSA J.

[id_DUK_389] De Santis Barbara, Raggi Maria, Moretti Giorgio, Facchiano Francesco, Mezzelani Alessandra, Villa Laura, Bonfanti Arianna, Campioni Alessandra, Rossi Stefania, Camposeo Serena, Soricelli Sabina, Moracci Gabriele, Debegnach Francesca, Gregori Emanuela, Ciceri Francesca, Milanesi Luciano, Marabotti Anna, Brera Carlo (2017). Study on the association among mycotoxins and other variables in children with autism. Toxins (Basel).

[id_DUK_390] Degen G. H., Blaskewicz M., Lektarau Y., Grüner C. (2003). Ochratoxin A Analysen im Blut vonArbeitnehmern in der Abfallwirtschaft. Mycotoxin Res.

[id_DUK_391] Degen Gisela H., Ali Nurshad, Gundert-Remy Ursula (2018). Preliminary data on citrinin kinetics in humans and their use to estimate citrinin exposure based on biomarkers. Toxicol Lett.

[id_DUK_392] Degen Gisela H., Reinders Jörg, Kraft Martin, Völkel Wolfgang, Gerull Felicia, Burghardt Rafael, Sievering Silvia, Engelmann Jennifer, Chovolou Yvonni, Hengstler Jan G., Fromme Hermann (2022). Citrinin exposure in Germany: urine biomarker analysis in children and adults. Toxins (Basel).

[id_DUK_393] Dohnal Vlastimil, Wu Qinghua, Kuča Kamil (2014). Metabolism of aflatoxins: key enzymes and interindividual as well as interspecies differences. Arch Toxicol.

[id_DUK_394] Duarte Sofia Cancela, Pena Angelina, Lino Celeste Matos (2011). Human ochratoxin A biomarkers—from exposure to effect. Crit Rev Toxicol.

[id_DUK_395] European Food Safety AuthorityEFSA (2006). Opinion of the Scientific Panel on Contaminants in the Food Chain on a request from the Commission related to ochratoxin A in food. EFSA J.

[id_DUK_396] Europäische Kommission (2002). Entscheidung der Kommission vom 12. August 2002 zur Umsetzung der Richtlinie 96/23/EG des Rates betreffend die Durchführung von Analysemethoden und die Auswertung von Ergebnissen (2002/657/EG). ABl L.

[id_DUK_397] Ferri Fulvio, Brera Carlo, De Santis Barbara, Fedrizzi Giorgio, Bacci Tiziana, Bedogni Lorena, Capanni Sauro, Collini Giorgia, Crespi Enrica, Debegnach Francesca, Ferdenzi Patrizia, Gargano Angelo, Gattei Daniela, Luberto Ferdinando, Magnani Ines, Magnani Massimo, Mancuso Pamela, Menotta Simonetta, Mozzanica Stefania, Olmi Milva, Ombrini Giuseppe, Sala Orietta, Soricelli Sabina, Vicentini Massimo, Giorgi Rossi Paolo (2017). Survey on urinary levels of aflatoxins in professionally exposed workers. Toxins (Basel).

[id_DUK_398] Fischer Guido, Müller Thomas, Schwalbe Regina, Ostrowski René, Dott Wolfgang (2000). Exposure to airborne fungi, MVOC and mycotoxins in biowaste-handling facilities. Int J Hyg Environ Health.

[id_DUK_399] Föllmann Wolfram, Ali Nurshad, Blaszkewicz Meinolf, Degen Gisela H. (2016). Biomonitoring of mycotoxins in urine: pilot study in mill workers. J Toxicol Environ Health A.

[id_DUK_400] Fromme H., Gareis M., Völkel W., Gottschalk C. (2016). Overall internal exposure to mycotoxins and their occurrence in occupational and residential settings – an overview. Int J Hyg Environ Health.

[id_DUK_401] Gao Shunxiang, Zheng Xin, Tang Yuan, Cheng Yajun, Hu Xiaobo, Wu Jihong (2019). Development of a fluorescently labeled aptamer structure-switching assay for sensitive and rapid detection of gliotoxin. Anal Chem.

[id_DUK_402] Gerding Johannes, Ali Nurshad, Schwartzbord Jeremy, Cramer Benedikt, Brown Dan L., Degen Gisela H., Humpf Hans-Ulrich (2015). A comparative study of the human urinary mycotoxin excretion patterns in Bangladesh, Germany, and Haiti using a rapid and sensitive LC-MS/MS approach. Mycotoxin Res.

[id_DUK_403] Greim H. (2003). Gesundheitsschädliche Arbeitsstoffe, Toxikologisch-arbeitsmedizinische Begründung von MAK-Werten.

[id_DUK_404] Greim H. (2008). Gesundheitsschädliche Arbeitsstoffe, Toxikologisch-arbeitsmedizinische Begründung von MAK-Werten.

[id_DUK_405] Hagelberg Sigrid, Hult Karl, Fuchs Radovan (1989). Toxicokinetics of ochratoxin A in several species and its plasma-binding properties. J Appl Toxicol.

[id_DUK_406] Kommission "Human-Biomonitoring" des UmweltbundesamtsHBM-Kommission (2014). Stoffmonographie Ochratoxin A. Bundesgesundheitsblatt Gesundheitsforschung Gesundheitsschutz.

[id_DUK_407] Hof Herbert, Kupfahl Claudio (2009). Gliotoxin in Aspergillus fumigatus: an example that mycotoxins are potential virulence factors. Mycotoxin Res.

[id_DUK_408] International Agency for Research on CancerIARC (1986). Some naturally occurring and synthetic food components, furocoumarins and ultraviolet radiation.

[id_DUK_409] Jubert Carole, Mata John, Bench Graham, Dashwood Roderick, Pereira Cliff, Tracewell William, Turteltaub Kenneth, Williams David, Bailey George (2009). Effects of chlorophyll and chlorophyllin on low-dose aflatoxin B₁ pharmacokinetics in human volunteers. Cancer Prev Res (Phila).

[id_DUK_410] Lanier Caroline, Richard Estelle, Heutte Natacha, Picquet Rachel, Bouchart Valérie, Garon David (2010). Airborne molds and mycotoxins associated with handling of corn silage and oilseed cakes in agricultural environment. Atmos Environ.

[id_DUK_411] Martins C., Assunção R., Nunes C., Torres D., Alvito P. (2021). Are data from mycotoxins’ urinary biomarkers and food surveys linked? A review underneath risk assessment. Food Rev Int.

[id_DUK_412] Muñoz Katherine, Blaszkewicz Meinolf, Degen Gisela H. (2010). Simultaneous analysis of ochratoxin A and its major metabolite ochratoxin alpha in plasma and urine for an advanced biomonitoring of the mycotoxin. J Chromatogr B Analyt Technol Biomed Life Sci.

[id_DUK_413] Nieminen Susanna M., Kärki Riikka, Auriola Seppo, Toivola Mika, Laatsch Hartmut, Laatikainen Reino, Hyvärinen Anne, von Wright Atte (2002). Isolation and identification of Aspergillus fumigatus mycotoxins on growth medium and some building materials. Appl Environ Microbiol.

[id_DUK_414] Nieminen Susanna M, Mäki-Paakkanen Jorma, Hirvonen Maija-Riitta, Roponen Marjut, von Wright Atte (2002). Genotoxicity of gliotoxin, a secondary metabolite of Aspergillus fumigatus, in a battery of short-term test systems. Mutat Res.

[id_DUK_415] Njumbe Ediage Emmanuel, Diana Di Mavungu Jose, Song Suquan, Wu Aibo, Van Peteghem Carlos, De Saeger Sarah (2012). A direct assessment of mycotoxin biomarkers in human urine samples by liquid chromatography tandem mass spectrometry. Anal Chim Acta.

[id_DUK_416] Penczynski Katharina J., Cramer Benedikt, Dietrich Stefan, Humpf Hans-Ulrich, Abraham Klaus, Weikert Cornelia (2022). Mycotoxins in serum and 24-h urine of vegans and omnivores from the risks and benefits of a vegan diet (RBVD) study. Mol Nutr Food Res.

[id_DUK_417] Scharf Daniel H., Brakhage Axel A., Mukherjee Prasun K. (2016). Gliotoxin – bane or boon?. Environ Microbiol.

[id_DUK_418] Schmidt Jessica, Cramer Benedikt, Turner Paul C., Stoltzfus Rebecca J., Humphrey Jean H., Smith Laura E., Humpf Hans-Ulrich (2021). Determination of urinary mycotoxin biomarkers using a sensitive online solid phase extraction-UHPLC-MS/MS method. Toxins (Basel).

[id_DUK_419] Studer-Rohr I., Schlatter Josef, Dietrich Daniel R. (2000). Kinetic parameters and intraindividual fluctuations of ochratoxin A plasma levels in humans. Arch Toxicol.

[id_DUK_420] Tangni Emmanuel K., Pussemier Luc (2007). Ergosterol and mycotoxins in grain dusts from fourteen Belgian cereal storages: a preliminary screening survey. J Sci Food Agric.

[id_DUK_421] Tao Yanfei, Xie Shuyu, Xu Fanfan, Liu Aimei, Wang Yanxin, Chen Dongmei, Pan Yuanhu, Huang Lingli, Peng Dapeng, Wang Xu, Yuan Zonghui (2018). Ochratoxin A: toxicity, oxidative stress and metabolism. Food Chem Toxicol.

[id_DUK_422] Viegas S., Veiga L., Figueredo P., Almeida A., Carolino E., Sabino R., Veríssimo C., Viegas C. (2013). Occupational exposure to aflatoxin B₁: the case of poultry and swine production. World Mycotoxin J.

[id_DUK_423] Viegas Susana, Veiga Luisa, Figueiredo Paula, Almeida Ana, Carolino Elisabete, Viegas Carla (2015). Assessment of workers’ exposure to aflatoxin B1 in a Portuguese waste industry. Ann Occup Hyg.

[id_DUK_424] Viegas Susana, Assunção Ricardo, Nunes Carla, Osteresch Bernd, Twarużek Magdalena, Kosicki Robert, Grajewski Jan, Martins Carla, Alvito Paula, Almeida Ana, Viegas Carla (2018). Exposure assessment to mycotoxins in a Portuguese fresh bread dough company by using a multi-biomarker approach. Toxins (Basel).

[id_DUK_425] Viegas Susana, Osteresch Bernd, Almeida Ana, Cramer Benedikt, Humpf Hans-Ulrich, Viegas Carla (2018). Enniatin B and ochratoxin A in the blood serum of workers from the waste management setting. Mycotoxin Res.

[id_DUK_426] Viegas Carla, Faria Tiago, Caetano Liliana Aranha, Carolino Elisabete, Quintal-Gomes Anita, Twarużek Magdalena, Kosicki Robert, Viegas Susana (2019). Characterization of occupational exposure to fungal burden in Portuguese bakeries. Microorganisms.

